# Assessing the robustness of sisVIVE in a Mendelian randomization study to estimate the causal effect of body mass index on income using multiple SNPs from understanding society

**DOI:** 10.1002/sim.8066

**Published:** 2018-12-18

**Authors:** Yanchun Bao, Paul S. Clarke, Melissa Smart, Meena Kumari

**Affiliations:** ^1^ Institute for Social and Economic Research University of Essex Colchester UK

**Keywords:** instrumental variables, Mendelian randomization, MR‐Egger, MR‐Median, pleiotropic bias, sisVIVE

## Abstract

The “some invalid, some valid instrumental variable estimator” (sisVIVE) is a lasso‐based method for instrumental variables (IVs) regression of outcome on an exposure. In principle, sisVIVE is robust to some of the IVs in the analysis being invalid, in the sense of being related to the outcome variable through pathways not mediated by the exposure. In this paper, we consider the application of sisVIVE to a Mendelian randomization study in which multiple genetic variants are used as IVs to estimate the causal effect of body mass index on personal income in the presence of unobserved confounding. In addition to analyzing data from the large‐scale longitudinal household survey Understanding Society, we conduct a simulation study to (a) assess the performance of sisVIVE in relation to that of competing robust methods like “MR‐Egger” and “MR‐Median” and (b) identify scenarios under which its absolute performance is poor. We find that sisVIVE outperforms alternative robust methods, in terms of mean‐square error, across a wide range of scenarios, but that its performance is poor in absolute terms when the presence of indirect pleiotropy leads to failure of the “InSIDE” condition, which is not explicitly required for identification. We argue that this is because the consistency criterion for sisVIVE does not identify the true causal effect when InSIDE fails.

## 1 INTRODUCTION

The some invalid, some valid instrumental variables estimator (sisVIVE) is a variant of the lasso for instrumental variables (IVs) regression when some of the IVs in the analysis are not valid.^1^ IV methods are used for Mendelian randomization (MR) studies in epidemiology^2^, ^3^ and social and economic research.^4^, ^5^ These studies involve the use of genetic variants as IVs to estimate the causal effects of modifiable exposures on outcomes from observational data. IVs can potentially overcome the problems of unobserved confounding and reverse causation that usually affect the estimation of causal effects using observational data. A genetic variant is a valid IV if it is (a) associated with the exposure, (b) has no direct effect on the outcome, and (c) has no indirect effect through the unobserved confounding variables on the outcome.

The genetic variants used in MR studies are called single‐nucleotide polymorphisms (SNPs). SNPs are locations in the DNA sequence that typically comprise a base pair of variant forms called alleles. The alleles at these locations vary between individuals in that individuals can have zero, one or two copies of a specific allele. If the exposure being studied is the expression (or phenotype) of a SNP, then this SNP is, potentially, a valid IV. If only one SNP is used as an IV, the causal effect of the exposure on the outcome is estimated using the ratio of the estimated coefficient of the SNP—obtained from the regression of the outcome on the SNP—to the corresponding coefficient from the regression of the exposure on the SNP.^6^ If the SNP is a valid IV, and the causal relationship between exposure and outcome is linear, the ratio estimator will be consistent, but not unbiased, for the true causal effect.

A well‐known problem with ratio estimators is “weak instrument bias,” that is, the bias that arises when an IV is insufficiently predictive of the exposure.^7^, ^8^ This is often true for MR studies where the correlations between SNPs and exposures tend to be small. One strategy for avoiding weak instrument bias is, if available, to use more than one SNP. Multiple SNPs can be combined into what are called allele, genetic or polygenic risk scores^9^ to be used as IVs for ratio estimation. Alternatively, one can use two‐stage least squares (2SLS), rather than ratio estimation, with the SNPs as multiple IVs.^10^ In both cases, the rationale for using multiple SNPs is that the additional information contained by these SNPs will predict the exposure more accurately and thus alleviate weak instrument bias.

In this paper, we use MR to estimate the causal effect of body mass index (BMI) on personal income using data from *Understanding Society*: *the UK Longitudinal Household Study* (UKHLS). UKHLS contains rich genetic data from which we obtain 71 out of the 97 common genetic variants found to be associated with BMI in genome‐wide association studies (GWASs) at the genome‐wide level of significance.^11^ We adopt the multiple SNPs strategy here because the single genetic marker most strongly associated with BMI—the FTO gene (rs1558902)—explains only 0.27% of the variation.

The use of multiple SNPs presents two further challenges. The first is that, even if all the SNPs were valid IVs, the combined explanatory power of the additional SNPs could still be small enough to lead to *many* weak instruments bias.^12^ This is certainly possible here because combining all 97 BMI‐associated SNPs from the GIANT study explains only 2.7% of the total variation in BMI.^11^ A “two‐sample” strategy has been proposed to alleviate this problem, that is, rather than estimating the SNP‐exposure associations using the analysis sample, estimates of these associations are taken from another, ideally much larger, data set.^13^ Provided that the second data set was drawn from the same (or at least a comparable) population as the original, and the estimates were precise, this strategy would eliminate weak instrument bias. Hence, we also consider a two‐sample strategy for our analysis using estimates of the associations between the SNPs and BMI from the GIANT consortium.^11^

The second, and most important, challenge facing any MR study is that some of the SNPs are not valid IVs satisfying conditions (a) to (c) above. This is usually caused by “pleiotropic” SNPs, that is, SNPs which are related to individual traits other than the exposure. However, there has been recent work on developing “robust” IV methods that adjust for pleiotropic bias. Our main methodological focus is thus on comparing the performance of the recently developed sisVIVE with two established approaches, “MR‐Egger” regression^14^ and “MR‐Median” regression.^15^ Our objectives are twofold: to assess the relative performance of sisVIVE in comparison with its competitors and to investigate scenarios under which sisVIVE performs poorly in absolute terms.

## 2 MODELS FOR MENDELIAN RANDOMIZATION STUDIES

### 2.1 Choosing SNPs

A single‐SNP MR study will typically involve a SNP for which there is robust evidence that it is a genotype for the exposure. This evidence is usually obtained from a dedicated GWAS. A GWAS involves estimating the associations between a genome‐wide set of genetic variants (typically SNPs) and biological traits to identify which of these variants are associated with each trait. GWAS are adjusted for errors due to multiple testing, potential confounders of these associations, and for population groups with different genotype distributions (the so‐called population stratification). The genome‐wide level of significance is determined statistically; a p‐value significance threshold of 5 × 10^−8^ has become widely accepted.

The accuracy of a GWAS in determining which SNPs are associated with a given trait depends on the sample size and, particularly, on the adequacy of the confounding and population‐stratification adjustments. However, as we have already discussed, there is also a risk that GWAS‐identified SNPs will be pleiotropic so that either condition (b) or (c) does not hold; this would lead to biased estimates even if the association between the SNP and exposure were not weak.^16^ In the subsequent development, we distinguish between the “direct” pleiotropy that results from failure of condition (b) and the “indirect” pleiotropy that results from failure of condition (c).

As explained above, the UKHLS contains 71 of the 97 SNPs identified by the GIANT consortium as being associated with BMI.^11^ The full list of these SNPs is given in Table S1 in the Supplementary Information; the distributions of each SNP in UKHLS and GIANT consortium are given in the Table.

### 2.2 Modeling assumptions

We denote the outcome variable by *Y* and the exposure by *X*, and consider scenarios in which *Y* can be treated as a continuous variable that is causally related to *X* by the linear model

(1) $$Y=\gamma_{0}+\gamma_{X}X+\epsilon_{Y},$$

where *γ*_*X*_ is the causal exposure effect, and *ϵ*_*Y*_ is the model error comprising the combined effect of every influence (apart from *X*) on outcome *Y*. Any adjustments for observed confounding variables associated with exposure and outcome may be included but, for notational simplicity, we have omitted these from (1). It is supposed either way that there remains substantial unobserved confounding because important confounding variables have been omitted. These variables are hence absorbed into *ϵ*_*Y*_ and lead to an association between *ϵ*_*Y*_ and *X*. In such cases, standard regression estimation of (1) using ordinary least squares (OLS) or generalized least squares would be inconsistent and biased for causal parameters like *γ*_*X*_.

The precise interpretation of *γ*_*X*_ depends on the assumptions we make about exposure‐effect heterogeneity, that is, between‐person variation in the causal effect of BMI on personal income. The estimate of *γ*_*X*_ can be interpreted as the average causal effect of BMI on personal income if (i) the effect of BMI on personal income is the exactly the same for everyone or, more realistically, (ii) the between‐person variation in the effect of BMI among people with the same observed BMI is the same for all levels of BMI (after adjusting for the multiple SNPs); see chapter 5.2 in the work of Wooldridge.^17^

MR studies involve choosing one or more SNPs to use as IVs. Suppose that we use GWAS studies to identify *J* SNPs, which we denote by **G** = (*G*_1_, …, *G*_*J*_). Each SNP takes values from {0, 1, 2} depending on the number of times that the allele associated with increased exposure was found at this gene location. This allele is referred to as the “risk” or “effect” allele, and the other as the “base” or “noneffect” allele. We choose the effect allele for each of the 71 SNPs to be the one used by the GIANT consortium; see Table S1 in the Supplementary Information.

Using this notation, we can respectively rewrite conditions (a) to (c) for a single SNP, *G*_*j*_, in a slightly more formal way as follows. (i) The exposure is associated with the SNP such that $E(Y|G_{j}=g)$ depends nontrivially on g. (ii) No direct pleiotropy corresponds to no direct effect of the SNP on the outcome such that $E(Y|X=x,G_{j}=g,\epsilon_{Y})$ does not depend on g. (iii) No indirect pleiotropy corresponds to no association between the SNP and the unobserved confounding variables such that $E(\epsilon_{Y}|G_{j}=g)=0$. Note that conditioning on the other SNPs, and on observed confounding variables, is suppressed. Conditions (i) to (iii) are sometimes referred to as the core conditions^10^; the SNP *G*_*j*_ is a valid IV only if it satisfies all three core conditions.

Finally, we take the *independence assumption* to hold throughout the following development, that is, the multiple SNPs are drawn from distinct gene regions such that the *G*_*j*_ are mutually independent at the population level.

## 3 ESTIMATING CAUSAL EXPOSURE EFFECTS

### 3.1 Estimation with multiple SNPs that are all valid IVs

*The ratio estimator and two‐stage least squares*: If only one valid SNP, *G*_*j*_, was available, then the ratio estimator

(2) $${\hat{\gamma }}_{X;j}={\hat{\Gamma }}_{j}/{\hat{b}}_{j}$$

would be consistent and efficient for the causal effect *γ*_*X*_. The numerator ${\hat{\Gamma }}_{j}$ is the OLS estimator of the coefficient of *G*_*j*_ in the regression of *Y* on *G*_*j*_, and the denominator ${\hat{b}}_{j}$ is the OLS estimator of the corresponding coefficient in the regression of *X* on *G*_*j*_. (Note that, if covariates were also included in this analysis, *Y* and *X* in the preceding description would be respectively replaced by the residuals obtained from regressing *Y* and then *X* on these covariates.)

The ratio estimator is a special case of 2SLS. 2SLS is consistent and normally distributed in large samples if all of the SNPs are valid IVs satisfying the core conditions. Stage one of 2SLS for a single exposure *X* involves fitting a regression model of the form

(3) $$X=\mathrm{zb}+\epsilon_{X}$$

using OLS to obtain $\hat{b}$, where **z** is a row vector of predictors comprising a constant term and the instrumental variable(s), **b** is a column vector of regression coefficients, and *ϵ*_*X*_ is the model residual satisfying *E*(*ϵ*_*X*_|**G**) = 0. Stage two involves using $\hat{b}$ to obtain $\hat{X}=z\hat{b}$ and then finally regressing *Y* on $\hat{X}$ using OLS; the 2SLS estimator ${\hat{\gamma }}_{X}^{2\mathrm{SLS}}$ is simply the OLS estimator of the coefficient of $\hat{X}$. Note that ${\hat{\gamma }}_{X}^{2\mathrm{SLS}}$ is equal to ${\hat{\gamma }}_{X;j}$ in (2) if **z** = (1, *G*_*j*_).

To facilitate our comparison with sisVIVE further on, we write the 2SLS estimator as

(4) $${\hat{\gamma }}^{2\mathrm{SLS}}=\underset{\gamma }{\mathrm{argmin}}\left[\left\{\varepsilon_{Y}\left(\gamma \right)Z\right\}W_{N}{\left\{\varepsilon_{Y}\left(\gamma \right)Z\right\}}^{\prime }\right],$$

where ${\hat{\gamma }}^{2\mathrm{SLS}}={\left({\hat{\gamma }}_{0}^{2\text{SLS}},{\hat{\gamma }}_{X}^{2\text{SLS}}\right)}^{\prime }$ is the 2SLS estimator of **γ** = (*γ*_0_, *γ*_*X*_)′, *Z* is the design matrix of candidate IVs with row *i* given by **z**_*i*_, *W*_*N*_ = (*Z*′*Z*)^−1^ is the weight matrix, **ε**_*Y*_(**γ**) = (*ε*_*Y*; 1_,…,*ε*_*Y*; *N*_) is the row vector of residuals *ε*_*Y*; *i*_ = *Y*_*i*_ − *γ*_0_ − *γ*_*X*_*X*_*i*_ under model (1), and **z**_*i*_, *X*_*i*_, and *Y*_*i*_ are respectively the observed values of the IVs, exposure, and outcome for individual *i* = 1,…, *N*.

There are thus two ways to incorporate multiple SNPs into an analysis.

*Polygenic risk scores*: The most straightforward strategy for IV analysis with multiple SNPs is to combine the SNPs to form a polygenic risk score (also known as an allele score or genetic risk score). The ratio estimator using the polygenic risk score as an IV can then be used to estimate *γ*_*X*_.^9^ A polygenic risk score has the general form

(5) $$G=\sum_{j=1}^{J}w_{j}G_{j},$$

where *w*_*j*_ is a user‐specified weight for SNP *G*_*j*_. The simple polygenic risk score (SPRS) is obtained by setting *w*_*j*_ = 1 for every SNP. Alternatively, the internally weighted polygenic risk score (IPRS) can be obtained by setting $w_{j}={\hat{b}}_{j}$. In either case, the ratio estimator with polygenic score *G* as the IV can be used to estimate the causal effect, or 2SLS with **z** = (1, *G*) in (2) can equivalently be used.

*Multiple IVs*: An alternative to polygenic risk scores is to treat the *J* SNPs as multiple IVs for exposure *X* by setting **z** = (1, **G**) in (2).

Bowden et al^14^ show that the ratio estimate based on a polygenic risk score *G* is a weighted sum of the ${\hat{\gamma }}_{X;j}$, that is, the ratio estimate obtained using SNP *j* as the sole IV for *X*; the SPRS and IPRS estimates differ in how the SNP‐specific estimates are combined. Multiple‐IV 2SLS is exactly equivalent to IPRS in large samples if the independence assumption holds, that is, the population correlations between the SNPs and exposure are all equal to zero. IPRS is the basis of the inverse weighted (IVW) estimator for MR studies using summarized data.^14^, ^18^

### 3.2 Estimation with multiple SNPs where some are invalid IVs using sisVIVE

If any of the chosen SNPs were invalid IVs, then every estimator discussed in Section 3.1 would be inconsistent and subject to further bias regardless of whether or not the IVs were weak. This is because pleiotropy would lead to model (1) being incorrectly specified. In the presence of pleiotropy, it is assumed that model (1) becomes

(6) $$Y=\pi_{0}+\gamma_{X}X+\sum_{j=1}^{J}\left(\alpha_{j}+\theta_{j}\right)G_{j}+\epsilon_{Y},$$

where model error *ϵ*_*Y*_ satisfies *E*(*ϵ*_*Y*_|**G**) = 0, *α*_*j*_ ≠ 0 if pleiotropic *G*_*j*_ fails core condition (ii), and *θ*_*j*_ ≠ 0 if it fails core condition (iii).^14^ Without any loss of generality, we shall henceforth assume that *Y*, *X*, and **G** are all mean centered so that *π*_0_ = 0.

Under model (6), the relationship between the true numerators and denominators of the SNP‐specific ratio estimators can be written as

(7) $$\Gamma_{j}=\pi_{j}+\gamma_{X}b_{j},$$

where *π*_*j*_ = *α*_*j*_ + *θ*_*j*_ is the sum of the pleiotropic errors related to SNP *j* = 1,…, *J*. This relationship drives the “consistency criterion” that identifies the causal exposure effect when the identity of the invalid‐IV SNPs is unknown.^1^

Kang et al^1^ developed sisVIVE (the “some invalid some valid instrumental variable estimator”) to estimate γ_*X*_ under model (4) when the set of valid‐IV SNPs is unknown. They first show that, while model (6) is not generally identified if the set of valid‐IV SNPs is unknown, it can be identified if over half of the SNPs are valid IVs, that is, the “majority rule.” Constrained estimation of model (6) subject to the majority rule holding is infeasible, but the closely related problem

(8) $$\left(\begin{array}{c} {\hat{\gamma }}_{X;\lambda }\\ {\hat{\pi }}_{\lambda } \end{array}\right)=\underset{\gamma ,\pi }{\mathrm{argmin}}\left[\frac{1}{2}\left\{\varepsilon_{Y}\left(\gamma_{X},\pi \right)Z\right\}W_{N}{\left\{\varepsilon_{Y}\left(\gamma_{X},\pi \right)Z\right\}}^{\prime }+\lambda ∥\pi {∥}_{1}\right]$$

is feasible. The terms of (8) are defined as follows: *λ* is a scalar “tuning” parameter; $∥\pi {∥}_{1}=\sum_{j=1}^{J}\left|\pi_{j}\right|$ is the *ℓ*_1_‐norm; *W*_*N*_ is the same weight matrix as used for 2SLS (2); **ε**_*Y*_(*γ*_*X*_, **π**) = (*ε*_*Y*; 1_,…, *ε*_*Y*;*N*_); *ε*_*Y*;*i*_ = *Y*_*i*_ − γ_*X*_*X*_*i*_ − **π***′***z**_*i*_ is the value of the residuals in model (4) for individual *i*; and **π** = (*π*_1_,…, *π*_*J*_)*′*. If both core conditions (ii) and (iii) held, then Equation (8) would reduce to 2SLS (4) because **π** would equal zero.

The resulting procedure is closely related to the “lasso” a technique used to identify subsets of predictor variables with nonzero regression coefficients.^19^ The idea is to constrain the sum of the absolute values of {*π*_*j*_} so that the optimizer is forced to shrink toward zero those *π*_*j*_ that have least impact on the first component {**ε**_*Y*_(*γ*_*X*_, **π**)*Z*}*W*_*N*_{**ε**_*Y*_(*γ*_*X*_, ***π***)*Z*}′ in (8), that is, the valid‐IV SNPs. In doing this, sisVIVE identifies the set $\hat{V}=\left\{j:{\hat{\pi }}_{j;\lambda }=0\right\}$ as an estimate of the true set of valid‐IV SNPs *V* = { *j* : *π*_*j*_ = 0}.

The choice of tuning parameter is crucial: a large value of *λ* would force **π** to be heavily penalized unless it were very close to zero, which would result in every SNP being classed as a valid IV; conversely, a small value of *λ* would lead to every SNP being treated as an invalid IV. The choice of *λ* is made using cross‐validation to estimate the prediction error across a range of fixed tuning‐parameter values. Rather than choosing the value *λ* = *λ*_min_ that minimizes the cross‐validation error, *λ*_min_ is incremented by a value determined by the “one standard error” rule (the standard error being that of the average prediction errors across the different cross‐validation samples) in order to reduce the chance of incorrectly identifying invalid‐IV SNPs as valid IVs.^1^, ^19^ This last point is important because incorrectly treating invalid‐IV SNPs as valid IVs has been shown to lead to greater bias than incorrectly identifying valid‐IV SNPs as invalid‐IV ones.^1^

Belloni and Chernozhukov^20^ have shown that the shrinkage induced by standard lasso estimators exacerbates the bias of the model‐parameter estimate and that sisVIVE is more effective at identifying the set of valid‐IV SNPs, *V*, than estimating **π** and *γ*_*X*_. They hence proposed the use of “post‐lasso” estimators to reduce this bias. In the same spirit, Windmeijer et al^21^ considered post‐lasso estimators for sisVIVE in which only the SNPs identified as valid IVs in $\hat{V}$ are used as multiple IVs in 2SLS; they found the resulting estimator to be subject to considerably lower bias than sisVIVE. However, they also derive theoretical results that show that sisVIVE is not always consistent, in the sense that $\hat{V}↛V$ as *n* → ∞: in short, if the relative strengths of the valid‐IV and invalid‐IV SNPs do not satisfy the “irrepresentable condition,”^22^ then sisVIVE will not be consistent and can perform poorly.

Finally, we make the following note about sisVIVE in the presence of heterogeneous causal effects. In Section 2.2, it was noted for 2SLS that *γ*_*X*_ can be interpreted as the average causal effect if the effect of BMI is homogenous, or there is heterogeneity but the between‐individual variation in BMI effects is independent of the exposure given the genetic IVs. Kang et al^1^ stated that the same assumptions are required if we wish to interpret *γ*_*X*_ as the average causal effect when using sisVIVE. However, we show that a further constraint is required if model (6) holds because the potential dependence of the mean treatment effect on the SNPs can result in *π*_*j*_ ≠ 0 even if SNP *j* is a valid IV, that is, the between‐individual variation in BMI causal effects is mean independent of both exposure and the SNPs (see Section S2.1 in the Supplementary Information).

We now briefly outline the two established robust methods to be compared with sisVIVE.

*MR‐Egger regression*: Bowden et al^14^ developed MR‐Egger by adapting the Egger regression technique from the meta‐analysis literature to MR. MR‐Egger involves treating estimates $\left\{{\hat{\Gamma }}_{j},{\hat{b}}_{j}:j=1,\text{…},J\right\}$ as data when fitting the simple linear regression

(9) $${\hat{\Gamma }}_{j}=\gamma_{0}^{\mathrm{Egg}}+\gamma_{X}^{\mathrm{Egg}}{\hat{b}}_{j}+\epsilon_{j}^{\mathrm{Egg}},$$

where $\epsilon_{j}^{\mathrm{Egg}}$ is a residual assumed to satisfy $E\left(\epsilon_{j}^{\mathrm{Egg}}\left|b_{j}\right.\right)=0$ (noting that expectation here is with respect to the set of chosen SNPs). Model (9) can be fitted using the inverse of the estimated residual variance from the regression of *Y* on SNP *j* as weights to account for differential minor allele frequencies (MAFs) such that SNPs with low MAFs contribute little to the estimation of (9).

The requirement that $E\left(\epsilon_{j}^{\mathrm{Egg}}\left|b_{j}\right.\right)=0$ is called the “InSIDE” condition.^14^ If InSIDE holds, then $\gamma_{X}^{\mathrm{Egg}}=\gamma_{X}$ and the true intercept term $\gamma_{0}^{\mathrm{Egg}}=E\left(\pi_{j}\right)$ is such that $\;\gamma_{0}^{\mathrm{Egg}}=0$ if the SNPs are all valid IVs. InSIDE is thought to be plausible if core condition (ii) fails but is generally unrealistic if condition (iii) fails because failure leads to both *b*_*j*_ and $\epsilon_{j}^{\mathrm{Egg}}$ depending on *θ*_*j*_.^14^ MR‐Egger also requires that ${\hat{b}}_{j}$ is precisely estimated because otherwise $\gamma_{X}^{\mathrm{Egg}}$ will be biased toward zero (see also the discussion of the “no measurement error assumption” in Section 3.3).

*MR‐Median*: Bowden et al^15^ proposed MR‐Median as an alternative to MR‐Egger that, in theory, does not require the InSIDE condition to hold. The authors view MR‐Median as a practicable alternative to the sisVIVE estimator to be introduced next. The main strengths of MR‐Median are its simplicity and that it can be used if only summary data are available. It is simply the median of the SNP‐specific ratio estimates; in other words, letting $\left\{{\hat{\gamma }}_{X\left(\;j\right)}:j=1,\text{…},J\right\}$ be the ordered set of SNP‐specific ratio estimates (ie, such that ${\hat{\gamma }}_{X\left(j+1\right)}\geq {\hat{\gamma }}_{X\left(j\right)}$ for all *j* = 1, …, *J* − 1), then ${\hat{\gamma }}_{X}^{\mathrm{Med}}={\hat{\gamma }}_{X\left(J/2\right)}$ if *J* is even and ${\hat{\gamma }}_{X}^{\mathrm{Med}}=\left\{{\hat{\gamma }}_{X\left(J/2\right)}+{\hat{\gamma }}_{X\left(J/2+1\right)}\right\}/2$ if *J* is odd. MR‐Median is consistent for *γ*_*X*_ if the majority rule holds, that is, less than *J*/2, or 50%, of the SNPs are invalid IVs but can be biased and does not converge in distribution to a normal random variable.

### 3.3 Two‐sample strategies

In the discussion so far, it has been assumed that the analysis will be based on one individual‐level data set. It is, however, possible to adopt a two‐sample strategy if estimates of the SNP‐exposure associations are available from another, preferably much larger, study.^23^, ^24^, ^25^ We denote the estimates obtained from this second sample by $\;\left\{\tilde{b_{j}}:j=1,\text{…},J\right\}$.

Estimates from a second sample can be incorporated into the approaches described above as follows.

- Externally weighted polygenic risk score (EPRS): choose $w_{j}=\tilde{\beta_{j}}$ in (5) and denote the resulting estimate by ${\hat{\gamma }}^{\text{EPRS}}$.
- 2SLS: Use $\tilde{X}=z^{\prime }\tilde{\beta }$ in stage two rather than $\;\hat{X}$.
- MR‐Egger: Fit ${\hat{\Gamma }}_{j}=\gamma_{0}^{\mathrm{Egg}}+\gamma_{X}^{\mathrm{Egg}}{\tilde{b}}_{j}+\epsilon_{j}^{\mathrm{Egg}}$ rather than (9).
- MR‐Median: The median of $\left\{{\hat{\tilde{\gamma }}}_{X\left(j\right)}:j=1,\text{…},J\right\}$, where ${\hat{\tilde{\gamma }}}_{X;j}={\hat{\Gamma }}_{j}/\tilde{b_{j}}$.
- sisVIVE: Use $\varepsilon_{Y;i}=Y_{i}-\gamma_{X}{\tilde{X}}_{i}-\pi^{\prime }z_{i}$, where ${\tilde{X}}_{i}=z_{i}^{\prime }\tilde{b}$, and apply (8).

In terms of estimator performance, the impact of the usual weak instrument bias is ameliorated using ${\hat{\tilde{\gamma }}}_{X;j}={\hat{\Gamma }}_{j}/\tilde{b_{j}}$ rather than ${\hat{\gamma }}_{X;j}$ because, in contrast to ${\hat{b}}_{j}$ and ${\hat{\Gamma }}_{j}$, there is no association between ${\tilde{b}}_{j}$ and ${\hat{\Gamma }}_{j}$ (or this association is very weak if the two samples have some individuals in common)^13^; in fact, if ${\tilde{b}}_{j}=b_{j}$, then ${\hat{\tilde{\gamma }}}_{j}$ would be unbiased because it would reduce to an OLS estimate from the regression of *Y* on the known and unconfounded variable *G*_*j*_*b*_*j*_. More realistically, we require the standard error of ${\tilde{b}}_{j}$ to be small, and both samples to be drawn from the same population (or at least, from two different but homogeneous populations), for ${\tilde{b}}_{j}≃b_{j}$ to hold.^26^, ^27^ Such scenarios satisfy what is called the no measurement error (NOME) condition.^13^ However, if ${\tilde{b}}_{j}$ is not precisely estimated, then NOME will not hold. NOME is so‐called because the imprecision means that $\;{\tilde{b}}_{j}=b_{j}+\mu_{j}$, where *μ*_*j*_ behaves like mean‐zero measurement error; the estimate is hence subject to an attenuation bias toward zero if NOME fails. This second form of weak‐instrument bias can be viewed as a kind of conservative shrinkage, which is less harmful than the first because it makes it more difficult to reject the null hypothesis of no causal effect.

### 3.4 Nonlinear exposure effects

The development above focuses on linear models with linear exposure effects. If the true exposure effect is nonlinear, with a curvilinear or piecewise‐linear form, then the ratio estimator is no longer consistent for the average causal effect. In the valid‐IV case, 2SLS needs only a small modification to remain consistent: the first stage is unchanged with only the second stage requiring modification to match the nonlinear form of the exposure effect. If the effect of *X* is piecewise nonlinear, such that the exposure is replaced by dummy variables representing the interval in which the observed exposure lies, identification typically requires at least as many IVs as there are piecewise intervals.

The robust methods considered here are all built around model (1) with a single‐parameter linear exposure effect. Neither MR‐Egger nor MR‐Median can be straightforwardly used because both hinge on the ratio estimator, which derives from the linear structural model (1). sisVIVE is based around constrained 2SLS estimation and so, in principle, can be adapted to nonlinear exposure effects. However, care must be taken to establish identification because the results underpinning sisVIVE are all based on the single‐SNP ratio estimates.^1^

## 4 ANALYSIS USING UKHLS DATA

MR has been used recently to understand the mechanism of relationship of high BMI and disadvantage of social‐economic status, such as low income: Tyrrell et al^28^ used highly self‐selected UK Biobank data for their analysis, where income is reported at the household level, and Böckerman et al^29^ used a small sample for their analysis, which severely limits the power of that study; we refer the reader to these articles for a justification for the existence of a linear effect of BMI on income. We apply the methods introduced above to the larger and more representative UKHLS sample data to estimate the causal effect of BMI on personal income. For our multiple SNPs, we use 71 out of the 97 common genetic variants identified as being associated with BMI at a genome‐wide level of significance.^11^ The 16 excluded SNPs were either obtained from analyses involving only men, only women, or including non‐Europeans (6 SNPs), or based on imputed data found to be below the 0.9 threshold (10 SNPs).^11^ The remaining 71 variants explain 1.6% of the variation in BMI among UKHLS participants; the effect‐allele frequencies from both UKHLS and the GIANT consortium^11^ are listed in Supplementary Information Table S1.

UKHLS is an annual household‐based panel study that started collecting information about the social, economic, and health status of its participants in 2009. The data set used for our analysis is drawn from the General Population Sample (GPS) and the British Household Panel Survey (BHPS) arms of UKHLS (BHPS merged with wave two of UKHLS in 2010). UKHLS collected additional health information, including BMI and blood samples, at wave two (for GPS members) and wave three (for BHPS members). A total of 10 480 individuals were genotyped using the Infinium Human Core Exome Beadchip. After quality‐control steps, a sample of 9944 individuals was obtained from which a further 1104 individuals were excluded using the following criteria: genetic relatedness larger than 0.05% (*N* = 707); BMI greater than 60 kg/m^2^ (*N* = 8); and aged under 25 (*N* = 389). The number of cases with at least one wave of personal income and no missing values of BMI, SNPs and covariates is *N* = 8047.

The outcome is average annual personal income (API) for each individual taken over three consecutive waves starting from the wave at which the individual's health information was recorded. For individuals with one or two of these observations missing, we took the mean API over the available waves. We standardized both BMI and API, where API was standardized separately for the GPS and BHPS samples because mean API is higher in the BHPS than in GPS. To control for population stratification, we restricted our analysis to the white population and included the following baseline covariates **C**: age (at which BMI was measured), gender, and the first 20 genetic principal components to control for ancestry.^27^

We do not consider nonlinear effects of BMI in this analysis. Our preliminary analyses did not detect any nonlinear effects of BMI on income, using the semiparametric method proposed by Stanley and Burgess,^30^ so we are able to focus on the linear‐exposure case and compare the performance of the three robust methods discussed above.

**Figure 1:**
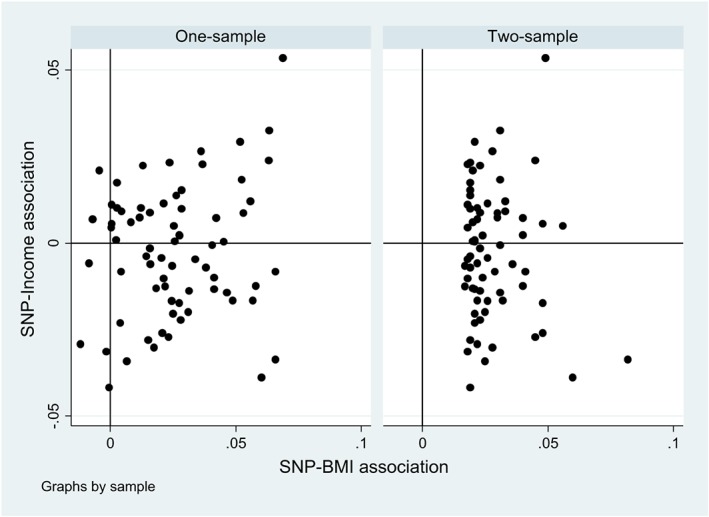
Scatterplot of SNP‐BMI association against SNP‐Income association of 71 SNPs under one‐sample and two‐sample strategies. Under one‐sample (left), both SNP‐BMI and SNP‐Income associations are from UKHLS, under two‐sample (right), SNP‐BMI associations are from GWAS study. BMI, body mass index; GWAS, genomewide association study; SNP, single‐nucleotide polymorphism; UKHLS, Understanding Society: the UK Longitudinal Household Study [Colour figure can be viewed at wileyonlinelibrary.com]

To obtain covariate‐adjusted estimates of the causal effect, we do not work with raw API and raw BMI but adjusted API and adjusted BMI. Each adjusted variable is equal to the fitted residual obtained from regressing it on **C**. For the two‐sample strategy, the exposure‐SNP estimates $\left\{{\tilde{b}}_{j}\right\}$ come from the GIANT study^11^ and so are already adjusted for **C**. The scatter plots of association of SNP‐BMI against associations of SNP‐Income for 71 SNPs based on one‐sample and two‐sample strategies are given in Figure 1.

It can be seen from Table 1 that the potentially confounded association between personal income and BMI has a significant negative value. Interpreting this estimate causally, we estimate that a one standard‐deviation increase in BMI (that is, of 5.26 BMI points) is estimated to lead on average to a decline in personal income of −0.032 of a standard deviation (£431.20).

**Table 1:** Estimates of the effect of BMI on personal income using n = 8047 cases from the UK Household Longitudinal Study

	Estimate	Std. Error	P‐value
**Observational association**	−0.032	0.011	0.002***
**One‐sample strategy**			
SPRS	−0.112	0.082	0.173
IPRS	−0.049	0.066	0.458
2SLS	−0.048	0.064	0.449
LIML	−0.058	0.081	0.470
Weighted MR‐Egger	−0.126	0.115	0.272
Egger test	0.003	0.004	0.411
Weighted MR‐Median	−0.137	0.097	0.157
sisVIVE	−0.048	‐	‐
Number of invalid IVs detected by sisVIVE	0		
**Two‐sample strategy**			
EPRS	−0.155	0.076	0.041**
2SLS	−0.154	0.071	0.030**
Weighted MR‐Egger	−0.311	0.179	0.082*
Egger test	0.005	0.005	0.302
Weighted MR‐Median	−0.292	0.106	0.006***
sisVIVE	−0.141	‐	‐
No. invalid IVs detected by sisVIVE	0		

Abbreviations: BMI, body mass index; EPRS, externally weighted polygenic risk score; IPRS, internally weighted polygenic risk score; IVs, instrumental variables; LIML, limited information maximum likelihood; MR, Mendelian randomization; sisVIVE, some invalid, some valid instrumental variable estimator; SPRS, simple polygenic risk score; 2SLS, two‐stage least squares.

Perhaps surprisingly, sisVIVE did not identify any invalid IVs. This is the case using both one‐sample and two‐sample strategies, and despite sisVIVE using the one‐standard‐error rule to choose the tuning parameter, which typically incorrectly identifies valid‐IV SNPs as invalid IVs.

We thus conclude that the valid‐IV methods discussed in Section 3.1 can be used in conjunction with all 71 SNPs to estimate the causal effect of BMI on personal income. The first pair of one‐sample results are ratio estimates obtained using for the SPRS and IPRS as IVs. The next two results use 2SLS and limited information maximum likelihood (LIML) treating the 71 SNPs as multiple distinct IVs. As expected, IRLS and multiple‐IV 2SLS give very similar results because the 71 SNPs are drawn from different gene regions and hence mutually independent. All four estimates are larger (negative) than the observational association but are also nonsignificant.

Because sisVIVE detects no invalid‐IV SNPs, the valid‐IV results above can also be classed as post‐sisVIVE estimates. We would reach the same conclusion using the other robust methods: the MR‐Egger test (for a nonzero intercept term) was not significant, and neither the MR‐Egger nor MR‐Median estimates were statistically significant (noting that the large standard errors indicate that both are less powerful than the valid‐IV methods when there are no invalid IVs).

Using a two‐sample strategy, we find no evidence for any invalid‐IVs SNPs but, in contrast to the one‐sample results, find a statistically significant large negative causal effect of BMI on personal income. The significance is, to a large extent, a consequence of estimated effect sizes that are larger than for any of the one‐sample results. Note also the following points: as expected, the EPRS and two‐sample version of multiple‐IV 2SLS produce very similar estimates because the 71 SNPs satisfy the independence assumption; and we do not present two‐sample LIML results because this estimator is used solely to reduce the impact of many weak instruments bias, which is not an issue for two‐sample strategies. The robust estimates obtained using MR‐Egger and MR‐Median are again far larger (negative) than the post‐sisVIVE ones but are statistically significant despite estimated less precisely.

The interpretation of these findings hinges crucially on the following untestable assumptions: the two‐sample approach requires that the data in the GIANT study and UKHLS are drawn from homogeneous populations; MR‐Median and sisVIVE both require that the total number of invalid‐IV SNPs does not exceed 35; and MR‐Egger requires that the InSIDE condition holds, that is, there is no relationship between the strength of SNP‐BMI association and the size of its pleiotropic effect on personal income in model (6). It is not possible to definitively resolve which of these assumptions holds, but in the next section, we conduct a simulation study to investigate more generally the relative performance of sisVIVE compared with other methods, for different numbers of invalid‐IV SNPs increases and different types of pleiotropy.

## 5 SIMULATION STUDY

We now carry out a simulation study to compare the effectiveness of sisVIVE with that of the other robust methods introduced above. The basic design of the study is based on those reported by Kang et al^1^ and Bowden et al,^14^ but here the data are simulated to mimic key characteristics of the UKHLS. Furthermore, the comparison is not just with 2SLS but with other robust methods.

We now set up a simulation study to investigate the following questions raised by the preceding development and data analysis:

- How accurately does sisVIVE identify valid‐IV SNPs and how is this accuracy affected as the number of valid‐IV SNPs increases?
- Post‐sisVIVE estimators involve using sisVIVE to identify the valid‐IV SNPs and then applying a valid‐IV estimator: in such cases, is 2SLS the best post‐sisVIVE estimator?
- Does the best post‐sisVIVE estimator perform better than MR‐Egger and MR‐Median in terms of estimator accuracy and confidence interval coverage?
- How well do post‐sisVIVE estimators perform in an absolute sense? Under which scenarios is its performance particularly good or particularly bad?

A final question, cutting across A to D, is the extent to which these answers depend on whether a one‐sample or two‐sample strategy is used.

A full description of the study design is given the Supplementary Information (Section S1) but we highlight some of its features here. The 71 SNPs *G*_1_, …, *G*_71_ are generated independently from a trinomial distribution in which the probabilities of *G*_*j*_ being 0, 1, and 2 are respectively equal to the proportions of *G*_*j*_ being 0, 1, and 2 in the UKHLS data (see Table S1 in the Supplementary Information). The causal association between BMI and SNP *j* is denoted by *β*_*j*_ and set to the value taken from the GIANT consortium study.^11^ In the one‐sample context, these are weak instruments because the average F‐statistic of the SNPs with respect to exposure BMI is 2.5 < 10. The true value of the causal effect of BMI on income is *γ*_*X*_ =  −0.2. The results presented below are all based on 1000 generated samples of size *N* = 10 000.

To simulate data subject to unobserved confounding, the error terms in personal‐income model (1) and BMI model (3) are respectively decomposed as $\epsilon_{Y}=U\gamma_{U}+{\dot{\epsilon }}_{Y}$ and $\epsilon_{X}=U+{\dot{\epsilon }}_{X}$, where *U* is a zero‐mean variable representing the unobserved confounding, and ${\dot{\epsilon }}_{Y}$ and ${\dot{\epsilon }}_{X}$ are not only mutually independent but jointly independent of (*Y*, *X*, **G**, *U*). Parameter *γ*_*U*_ = 1 controls the impact of unobserved confounding, that is, the strength and sign of the correlation between *ϵ*_*Y*_ and *ϵ*_*X*_, such that there is no unobserved confounding if *γ*_*U*_ = 0. Values of the outcome and exposure are respectively generated under model (1) and model (3), with $U,{\dot{\epsilon }}_{X}\;$and $\;{\dot{\epsilon }}_{Y}$ all independently generated from the standard normal distribution.

To generate pleiotropic SNPs, personal‐income outcomes are generated under model (6) under three different pleiotropy scenarios:

**Scenario 1:** Balanced direct pleiotropy where the InSIDE condition holds: *θ*_*j*_ = 0 for all *j* = 1, …, *J* but *α*_*s*_∼*U*(−0.2,0.2) (where *U*(*a*, *b*) indicates the continuous uniform distribution on real interval (*a*, *b*)) for SNPs *s* = 1, …, *S* ≤ *J*/2, and *α*_*j*_ = 0 for *j* = *S* + 1, …, *J*. Under this scenario, individual SNPs are pleiotropic but the (externally weighted) polygenic risk score is not because $E\left(\sum_{s=1}^{S}\alpha_{s}\right)=0$. The true SNP‐exposure association is equal to the causal effect of the SNP: *b*_*j*_ = *β*_*j*_.

**Scenario 2:** As Scenario 1 (including that *θ*_*j*_ = 0 for all SNPs *j* = 1, …, *J*, and *b*_*j*_ = *β*_*j*_), except that the direct pleiotropy is now unbalanced because *α*_*s*_∼*U*(0,0.2) and $E\left(\sum_{s=1}^{S}\alpha_{s}\right)>0$ for the pleiotropic/invalid‐IV SNPs.

**Scenario 3:** As Scenario 2, except that the pleiotropic SNPs are also indirectly pleiotropic with positive *θ*_*s*_∼*U*(0,0.4). The InSIDE assumption necessarily fails in this scenario because the true association between *G*_*s*_ and exposure is *b*_*s*_ = *β*_*s*_ + *θ*_*s*_ rather than *β*_*s*_ (in contrast, for the valid‐IV SNPs, *b*_*j*_ = *β*_*j*_) and so related to the pleiotropic effect.

For each of these scenarios, the performance of the estimators is assessed for *S* = 10, 20, and 30 invalid IVs, which respectively correspond to 14%, 28%, and 42% of the SNPs; these scenarios all satisfy the requirement that less than 50% of the SNPs are invalid.

For the two‐sample strategy, we consider three levels of accuracy for the estimates from the consortium study. The first accuracy level (“True”) is the gold standard in which ${\tilde{b}}_{j}$ is taken to equal the true association *b*_*j*_. The second level (“Precise”) has the association estimated with high accuracy ${\tilde{b}}_{j}∼N\left(b_{j},0.01^{2}\right)$. The third level (“Imprecise”) has ${\tilde{b}}_{j}∼N\left(b_{j},0.05^{2}\right)$ to represent situations where the consortium study yields noisy estimates. The precise and imprecise accuracy levels reflect degrees of failure of the NOME condition.^13^

Finally, the following *Stata* functions are used to implement the methods described above: ivreg2 to implement SPRS, EPRS, multiple‐IV 2SLS, and LIML; mregger from the mrrobust package to implement MR‐Egger regression; mrmedian from the mrrobust package for MR‐Median. The R package sisVIVE (downloaded from CRAN at https://cran.r-project.org/web/packages/sisVIVE.html) is used for sisVIVE.

*Question A: Selecting valid‐IV SNPs*: We first assess the performance of sisVIVE in terms of False Select In (FSI) and False Select Out (FSO). For each simulated data set, FSI is the ratio of the number of pleiotropic SNPs incorrectly identified as valid IVs to the total number of pleiotropic SNPs; and FSO is the ratio of valid‐IV SNPs incorrectly identified as invalid IVs to the total number of valid‐IV SNPs. The results that are shown in Table 2 are the mean FSI and mean FSO cross 1000 simulated data sets for each of the pleiotropy scenarios defined above.

**Table 2:** Mean FSI% and FSO% of sisVIVE for 1000 simulation data sets

		One Sample				Two Sample			
				True:		Precise:		Imprecise:	
				${\tilde{b}}_{j}=\beta_{j}$		${\tilde{b}}_{j}∼N\left(\beta_{j},0.01^{2}\right)$		${\tilde{b}}_{j}∼N\left(\beta_{j},0.05^{2}\right)$	
Scenarios	No. IVs	MFSI	MFSO	MFSI	MFSO	MFSI	MFSO	MFSI	MFSO
	Invalid	(%)	(%)	(%)	(%)	(%)	(%)	(%)	(%)
All IVs are valid	0	‐	6.9	‐	0	‐	0	‐	0.42
InSIDE holds and	10	30.1	2.9	29.9	1.7	30.3	1.8	30.0	2.1
Direct Pleiotropy	20	20.6	9.2	21.2	6.8	21.3	6.7	20.8	7.9
(balanced)	30	17.0	13.3	17.3	13.3	17.2	13.4	17.3	14.7
InSIDE holds and	10	36.1	1.9	30.5	2.4	31.0	2.2	31.6	2.2
Direct Pleiotropy	20	25.3	12.8	24.9	13.0	24.8	12.5	23.7	8.9
(positive)	30	21.2	31.9	23.3	32.3	23.0	30.4	21.0	18.6
InSIDE fails and	10	31.9	41.3	35.7	42.7	34.4	43.9	10.6	40.8
Direct Pleiotropy	20	39.2	42.1	32.5	54.2	32.6	54.2	23.1	66.3
(positive)	30	37.6	44.5	29.5	59.5	29.1	59.3	22.4	70.6

Abbreviations: IVs, instrumental variables; FSI, False Select In; FSO, False Select Out; MFSI, mean FSI; MFSO, mean FSO; sisVIVE, some invalid, some valid instrumental variable estimator.

Starting with FSO, when the number of pleiotropic SNPs is 30, sisVIVE incorrectly identifies (on average) up to 71% of the valid‐IV SNPs as invalid IVs, but it performs well (no more than 5% of valid‐IV SNPs incorrectly identified) when there are only 10 pleiotropic SNPs. The deterioration in FSO as the number of pleiotropic SNPs increases is apparent under both direct pleiotropy scenarios (Scenarios 1 and 2) for both one‐ and two‐sample strategies. The deterioration is also apparent under Scenario 3 (direct and indirect pleiotropy) but FSO exceeds 40% even if there are only 10 pleiotropic SNPs. The performance of sisVIVE in terms of FSO using a two‐sample strategy and using a one‐sample strategy are generally similar, except under the direct pleiotropy scenarios when the accuracy level of the SNP‐BMI associations is imprecise; in this case, failure of the NOME condition appears to prevent valid‐IV SNPs being incorrectly classed as invalid IVs.

In terms of FSI, between 39% and 11% of the invalid‐IV SNPs are incorrectly identified as valid IVs under the scenarios we consider. Superficially, sisVIVE performs best in terms of FSI for two‐sample strategies under Scenario 3 (direct and indirect pleiotropy) when the accuracy level of the SNP‐BMI associations is imprecise, but this is counteracted by its FSO performance being the worst among all the scenarios.

*Question B: Post‐sisVIVE estimators*: To assess the performance of alternative post‐sisVIVE estimators, we consider the “oracle” properties of the different valid‐IV estimators from Section 3.1 (and the two‐sample equivalents of these estimators in Section 3.3). In other words, we assess how well each valid‐IV estimator performs when the actual identities of the valid‐IV SNPs are known. The results are presented in the Supplementary Information (Table S2).

In summary, we found that the one‐sample versions of the valid‐IV estimators, except for SPRS and LIML, were adversely affected by many weak instruments bias.^12^ SPRS outperformed IPRS in terms of bias and MSE, despite IPRS having smaller standard errors. Only SPRS and LIML offer close‐to‐nominal coverage and were the most powerful at detecting the true causal effects. Using a two‐sample strategy, every estimator was nearly unbiased for the true and precise accuracy scenarios, but every method was found to be biased toward zero under the imprecise accuracy scenario, with the consequent impact on coverage and power. The exception was EPRS for which bias was less affected by the failure of the NOME condition than the other estimators, but its standard error was inflated leading to a high MSE and very low power.

We hence prefer SPRS as the post‐sisVIVE estimator when using a one‐sample strategy, and EPRS when using a two‐sample strategy. We also, however, present results from 2SLS in both cases for the purposes of comparison.

*Question C: Do the best post‐sisVIVE estimators perform better than MR‐Egger and MR‐Median?* Table 3 contains the results for scenarios with 10 invalid‐IV SNPs using one‐ and two‐sample strategies. Table 3a contains those results under pleiotropy Scenario 2 (unbalanced direct pleiotropy where InSIDE holds) and Table 3b those under Scenario 3 (indirect and unbalanced direct pleiotropy where InSIDE fails). For the one‐sample strategy, results are presented for MR‐Egger, MR‐Median, post‐sisVIVE SPRS, and post‐sisVIVE 2SLS. For the two‐sample strategy, results are presented for MR‐Egger, MR‐Median, post‐sisVIVE EPRS, and post‐sisVIVE 2SLS at the true accuracy level for the consortium estimates. The full set of results across all three pleiotropy scenarios for 10, 20, and 30 invalid IVs are presented in Tables S3 to S11 in the Supplementary Information.

**Table 3:** Simulation results for multiple instruments when 10 SNPs are invalid, U∼N(0, 1), ε_Xi_ ∼N(0, 1), ε_Yi_ ∼N(0, 1), MC step = 1000, and sample size = 10,000

(A)					
	10 Pleiotropic SNPs				
	(Positive Direct Pleiotropy, InSIDE Holds)				
True value	−0.2				
	Mean (SD)	Mean SE	MSE	Coverage, %	Power, %
**One‐sample strategy**					
SPRS	0.342 (0.145)	0.092	0.315	9.0	86.4
IPRS	0.270 (0.124)	0.150	0.236	7.0	42.5
Weighted Egger	0.199 (0.258)	0.257	0.225	64.8	13.3
Weighted Median	0.076 (0.110)	0.114	0.088	30.6	9.2
sisVIVE‐SPRS	−0.106 (0.115)	0.106	0.022	81.2	15.1
sisVIVE‐IPRS	0.042 (0.083)	0.078	0.065	14.5	11.6
**Two‐sample strategy, True precision** ${\tilde{b}}_{j}=\beta_{j}$					
EPRS	0.251 (0.136)	0.084	0.222	2.0	75.4
2SLS	0.255 (0.139)	0.192	0.226	24.7	15.3
Weighted Egger	−0.214 (0.483)	0.473	0.233	93.7	6.4
Weighted Median	−0.099 (0.154)	0.152	0.034	90.3	10.6
sisVIVE‐SPRS	−0.130 (0.118)	0.108	0.019	85.3	20.0
sisVIVE‐2SLS	−0.128 (0.109)	0.096	0.017	84.2	29.5
**(B)**					
	**10 Pleiotropic SNPs**				
	**(Positive Direct and Indirect Pleiotropy, InSIDE Fails)**				
	**Mean (SD)**	**Mean SE**	**MSE**	**Coverage, %**	**Power, %**
**One‐sample strategy**					
SPRS	0.574 (0.079)	0.047	0.606	0	100
IPRS	0.938 (0.078)	0.056	1.301	0	100
Weighted Egger	1.093 (0.097)	0.061	1.682	0	100
Weighted Median	0.998 (0.115)	0.065	1.449	0	100
sisVIVE‐SPRS	0.517 (0.145)	0.115	0.535	2.3	91.5
sisVIVE‐IPRS	0.761 (0.140)	0.058	0.944	0.1	99.4
**Two‐sample strategy, True precision** ${\tilde{b}}_{j}=\beta_{j}$					
EPRS	0.968 (0.080)	0.031	1.371	0	100
2SLS	0.970 (0.079)	0.057	1.376	0	100
Weighted Egger	1.149 (0.104)	0.057	1.830	0	100
Weighted Median	1.016 (0.116)	0.066	1.492	0	100
sisVIVE‐SPRS	0.550 (0.149)	0.077	0.585	0.1	98.8
sisVIVE‐2SLS	0.814 (0.133)	0.051	1.062	0	99.7

Abbreviations: EPRS, externally weighted polygenic risk score; IPRS, internally weighted polygenic risk score; SD, standard deviation; SE, standard error; sisVIVE, some invalid, some valid instrumental variable estimator; SPRS, simple polygenic risk score; 2SLS, two‐stage least squares.

The picture that emerges from these results is that, across these scenarios, sisVIVE SPRS has the best performance in terms of bias and MSE, closely followed by sisVIVE‐2SLS. If InSIDE holds, then MR‐Egger performs best in terms of bias and confidence interval coverage using a two‐sample strategy, but it performs considerably less well than post‐sisVIVE SPRS and 2SLS in terms of mean‐square error; this is also the case under the precise and imprecise accuracy levels (see Tables S6‐S8 in the Supplementary Information). The poor performance of MR‐Egger under the one‐sample strategy is thus explained by the impact of many weak instruments bias.

*Question D: How well does sisVIVE do in an absolute sense?* It is clear that, across all the scenarios, the post‐sisVIVE estimators are subject to substantial bias and that these biases are very large if (a) a one‐sample strategy is used (in all circumstances apart from the less plausible Scenario 1) and (b) Scenario 3 holds (where InSIDE fails due to the presence indirect pleiotropy). This conclusion holds if performance is also judged in terms of coverage and power of the normal‐based confidence intervals.

Overall, the same patterns are found for scenarios with 20 (see Tables S4, S7, and S10 in the Supplementary Information) and 30 (see Tables S5, S8, and S11) invalid‐IV SNPs, except that the magnitude of the biases and MSEs, and the extent to which the confidence intervals fail to achieve nominal coverage, becomes worse as the number of invalid‐IV SNPs increases.

The poor performance of the post‐sisVIVE estimators under Scenario 3 is surprising because, superficially at least, these require only that less than half of the SNPs are invalid. We investigate this further by rerunning the simulations for Scenario 3 but, when using a two‐sample strategy, took the true causal effects *β*_*j*_ of the SNPs on the exposure to be known to us for the pleiotropic SNPs rather than observed association *b*_*j*_ = *β*_*j*_ + *θ*_*j*_. The results (not presented) show that the bias, MSE, and coverage of the sisVIVE‐based approaches are similar to those under Scenarios 1 and 2; the same is also true for MR‐Median. This indicates that indirect pleiotropy and the failure of InSIDE have, contrary to theory, a detrimental effect on these methods' performance.

One possible explanation for this is set out by Windmeijer et al.^21^ Their proposition 2 contains conditions under which sisVIVE will not satisfy the “irrepresentable condition” of Zou^22^ and, hence, cannot be consistent. Crudely put, this condition states that sisVIVE will only be consistent if the strength of the invalid‐IV SNPs is greater than those of the valid‐IV ones. This is a possible explanation for the poor performance of sisVIVE in our simulations because our scenario 3 simulations do not satisfy it. However, using corollary 1,^21^ we set up a new version of our third scenario in which InSIDE fails but proposition 2 is satisfied; the new design and results are presented in the Supplementary Information: the design is described in Section S2.2, and the results for 10, 20, and 30 invalid‐IV SNPs respectively presented in Tables S12, S13, and S14. It can be seen from these results that sisVIVE performs as badly as it did when the irrepresentable condition fails, so we conclude that failure of the irrepresentable condition does not explain our results.

We instead argue that sisVIVE's poor performance is because the consistency criterion (defined in theorem 1)^1^, which underpins the identification sisVIVE, cannot be satisfied if indirect pleiotropy leads to failure of the InSIDE condition. In short, even were the identity of the valid‐IV and invalid‐IV SNPs known, identification would not be possible because the effects of direct pleiotropy—and hence, the combined pleiotropic effects—would be confounded with the invalid‐IV SNPs' causal effects on the exposure, so that the consistency criterion would not hold. This result also explains why the performance of sisVIVE improved in the hypothetical situation, explored above, where *β*_*j*_ (the causal effect of SNP on exposure) were known, because this identifies the combined pleiotropic effect. We provide a more detailed argument in Section S2.3 in the Supplementary Information.

## 6 DISCUSSION

Our investigation has revealed that, while it outperforms the alternative approaches we considered, sisVIVE performs well in terms of mean square error but performs particularly poorly if the InSIDE condition fails due to the presence of indirect pleiotropy. This is despite these scenarios apparently satisfying the sufficient condition that more than 50% of the SNPs are valid IVs. We argue that this is due to indirect pleiotropy leading to nonidentification and failure of the consistency criterion of Kang et al. The conclusion from our data analysis, which indicates the true causal effect of BMI could be underestimated by as much as a factor of five, is thus dependent on an assumption that there is no indirect pleiotropy through which the SNPs are related to the confounders. In general, such an occurrence cannot be ruled out because genes are determined at conception, and confounders determined at any point up to the time at which an individual's exposure is determined. The exception to this would be if there was strong scientific evidence that the phenotypical trait of each SNP was functionally related to the exposure of interest.

The performance of sisVIVE in scenarios where InSIDE holds and there is no indirect pleiotropy is more encouraging, but more generally, we would recommend that MR studies should be based on a sensitivity analysis involving robust MR‐Egger and “valid IV” methods to capture whether differences between the estimates point to the presence of pleiotropic SNPs. We would also concur with others who have suggest using unweighted polygenic risk scores or a two‐sample strategy or both.^31^, ^32^, ^33^ For a one‐sample strategy, it appears that imprecision in the estimated weights of an IPRS, while improving efficiency, can lead to substantial bias. While SPRSs perform well here, it is important to note that, in further simulations, we found (results not shown) that severe bias was introduced if the effect‐allele coding of the SNPs led to ${\hat{\beta }}_{j}$ (or $\;{\tilde{\beta }}_{j}$) being positive when true *β*_*j*_ would have led us to code it the other way; such “flip flopping” is possible,^34^ even in the absence of population stratification, but remains a potential source of bias for SPRS despite its being discounted elsewhere (eg, see page 1883 in the work of Burgess et al).^35^ The LIML or CUE estimators would, however, be unaffected by flip‐flopping and, unlike 2SLS, not subject to large biases.

## Supporting information

SIM_8066‐Supp‐0001‐Multiple_SNPs_Draft_StatMed_Revision_supplementary.docx

## References

[1] Kang H , Zhang A , Cai TT , Small DS . Instrumental variables estimation with some invalid instruments and its application to Mendelian randomization. JASA. 2016;111(513):132‐144.

[2] Lawlor DA , Harbord RM , Sterne JAC , Timpson N , Smith GD . Mendelian randomization: using genes as instruments for making causal inferences in epidemiology. Statist Med. 2008;27(8):1133‐1163. 10.1002/sim.3034 17886233

[3] Burgess S , Thompson SG . Mendelian Randomization—Methods for Using Genetic Variants in Causal Estimation. New York, NY: Chapman & Hall/CRC; 2014.

[4] Scholder SVK , Smith GD , Lawlor DA , Propper C , Windmeijer F . Child height, health and human capital: evidence using genetic markers. Eur Econ Rev. 2013;57:1‐22. 25673883 10.1016/j.euroecorev.2012.09.009 PMC4318168

[5] Tillmann T , Vaucher J , Okbay A , et al. Education and coronary heart disease: mendelian randomisation study. BMJ. 30 2017;358:j3542. 28855160 10.1136/bmj.j3542 PMC5594424

[6] Didelez V , Sheehan N . Mendelian randomization as an instrumental variable approach to causal inference. Stat Methods Med Res. 2007;16(4):309‐330. 17715159 10.1177/0962280206077743

[7] Burgess S , Thompson SG , CRP CHD Genetics Collaboration . Avoiding bias from weak instruments in Mendelian randomization studies. Int J Epidemiol. 2011;40(3):755‐764. 21414999 10.1093/ije/dyr036

[8] Stock J , Wright J , Yogo M . A survey of weak instruments and weak identification in generalized method of moments. J Bus Econ Stat. 2002;20:518‐529.

[9] Burgess S , Thompson SG . Use of allele scores as instrumental variables for Mendelian randomization. Int J Epidemiol. 2013;42(4):1134‐1144. 24062299 10.1093/ije/dyt093 PMC3780999

[10] Burgess S , Small DS , Thompson SG . A review of instrumental variable estimators for Mendelian randomization. Stat Methods Med Res. 2015;26(5):2333‐2355. 26282889 10.1177/0962280215597579 PMC5642006

[11] Locke AE , Kahali B , Berndt SI , et al. Genetic studies of body mass index yield new insights for obesity biology. Nature. 2015;518(7538):197‐206. 25673413 10.1038/nature14177 PMC4382211

[12] Davies NM , von Hinke Kessler Scholder S , Farbmacher H , Burgess S , Windmeijer F , Smith GD . The many weak instruments problem and Mendelian randomization. Statist Med. 2015;34(3):454‐468. 10.1002/sim.6358 PMC4305205 25382280

[13] Bowden J , Del Greco FM , Minelli C , Smith GD , Sheehan NA , Thompson JR . Assessing the suitability of summary data for two‐sample Mendelian randomization analyses using MR‐egger regression: the role of the I^2^ statistic. Int J Epidemiol. 2016;45(6):1961‐1974. 27616674 10.1093/ije/dyw220 PMC5446088

[14] Bowden J , Smith GD , Burgess S . Mendelian randomization with invalid instruments: effect estimation and bias detection through egger regression. Int J Epidemiol. 2015;44(2):512‐525. 26050253 10.1093/ije/dyv080 PMC4469799

[15] Bowden J , Smith GD , Haycock PC , Burgess S . Consistent estimation in Mendelian randomization with some invalid instruments using a weighted median estimator. Genet Epidemiol. 2016;40(4):304‐314. 27061298 10.1002/gepi.21965 PMC4849733

[16] Del Greco MF , Minelli C , Sheehan NA , Thompson JR . Detecting pleiotropy in Mendelian randomisation studies with summary data and a continuous outcome. Statist Med. 2015;34(21):2926‐2940. 10.1002/sim.6522 25950993

[17] Wooldridge JM . Econometric Analysis of Cross‐Sectional and Panel Data. 1st ed. Cambridge, MA: MIT Press; 2002.

[18] Burgess S , Bowden J . Integrating summarized data from multiple genetic variants in Mendelian randomization: bias and coverage properties of inverse‐variance weighted methods. 2015. arXiv preprint arXiv:1512.04486.

[19] Natarajan BK . Sparse approximate solutions to linear systems. SIAM J Comput. 1995;24:227‐234.

[20] Belloni A , Chernozhukov V . Least squares after model selection in high‐dimensional sparse models. Ther Ber. 2013;19(2):521‐547.

[21] Windmeijer F , Farbmacher H , Davies N , Smith GD . On the use of the Lasso for instrumental variables estimation with some invalid instruments. http://www.efm.bris.ac.uk/economics/working_papers/pdffiles/dp16674.pdf. 2017. 10.1080/01621459.2018.1498346 PMC6817329 31708716

[22] Zou H . The adaptive Lasso and its oracle properties. JASA. 2006;101(476):1418‐1429.

[23] Pierce B , Burgess S . Efficient design for Mendelian randomization studies: subsample and two‐sample instrumental variable estimators. Am J Epidemiol. 2013;178(7):1177‐1184. 23863760 10.1093/aje/kwt084 PMC3783091

[24] Angrist JD , Imbens GW . Two‐stage least squares estimation of average causal effects in models with variable treatment intensity. JASA. 1995;90(430):431‐442.

[25] Inoue A , Solon G . Two‐sample instrumental variables estimators. Rev Econ Stat. 2010;92:557‐561.

[26] Burgess S , Butterworth A , Thompson SG . Mendelian randomization analysis with multiple genetic variants using summarized data. Genet Epidemiol. 2013;37(7):658‐665. 24114802 10.1002/gepi.21758 PMC4377079

[27] Price AL , Patterson NJ , Plenge RM , Weinblatt ME , Shadick NA , Reich D . Principal components analysis corrects for stratification in genome‐wide association studies. Nat Genet. 2006;38(8):904‐909. 16862161 10.1038/ng1847

[28] Tyrrell J , Jones SE , Beaumont R , et al. Height, body mass index, and socioeconomic status: Mendelian randomisation study in UK Biobank. BMJ. 2016;352:i582 26956984 10.1136/bmj.i582 PMC4783516

[29] Böckerman P , Cawley J , Viinikainen J , et al. *The Effect of Weight on Labor Market Outcomes: An Application of Genetic Instrumental Variables* Paper Series. Cambridge, MA: National Bureau of Economic Research Working; 2016. 10.1002/hec.3828 PMC6585973 30240095

[30] Staley JR , Burgess S . Semiparametric methods for estimation of a nonlinear exposure‐outcome relationship using instrumental variables with application to Mendelian randomization. Genet Epidemiol. 2017;41:341‐352. 28317167 10.1002/gepi.22041 PMC5400068

[31] Hartwig F , Davies N . Why internal weights should be avoided (not only) in MR egger regression. Int J Epidemiol. 2016;45(5):1676‐1678. 27649799 10.1093/ije/dyw240

[32] Bowden J , Burgess S , Davey Smith G . Response to Hartwig and Davies. Int J Epidemiol. 2016;45(5):1679‐1680. 27649802 10.1093/ije/dyw252 PMC5100626

[33] Kemp FJ , Sayers A , Davey Smith G , Tobias JH , Evans DM . Authors' response to Hartwig and Davies. Int J Epidemiol. 2016;45(5):1678‐1679. 27649804 10.1093/ije/dyw241 PMC5100625

[34] Lin PI . No gene is an island: the flip‐flop phenomenon. Am J Hum Genet. 2007;80(5):1002‐1002. 10.1086/512133 PMC1821115 17273975

[35] Burgess S , Dudbridge F , Thompson SG . Combining information on multiple instrumental variables in Mendelian randomization: comparison of allele score and summarized data methods. Statist Med. 2016;35(11):1880‐1906. 10.1002/sim.6835 PMC4832315 26661904

