# Supplementary material for: Assessing the robustness of sisVIVE in a Mendelian randomization study to estimate the causal effect of body mass index on income using multiple SNPs from understanding society
Source: Stat Med. 2018 Dec 18;38(9):1529–42. doi: 10.1002/sim.8066 (PMC6492086; doi:10.1002/sim.8066)
Supplement: Supplementary file 1 — SIM_8066‐Supp‐0001‐Multiple_SNPs_Draft_StatMed_Revision_supplementary.docx [file SIM-38-1529-s001.docx]

**Assessing the robustness of sisVIVE in a Mendelian randomization study to estimate the causal effect of body mass index on income using multiple SNPs from Understanding Society (Supplementary)**

Yanchun Bao,^*^ Paul S Clarke, Melissa C Smart, Meena Kumari.

Supplementary section 1: Data design for simulation

All the results in simulation study are based on 1000 generated samples each of size$N=10 000$.

S1.1 All SNPs are invalid

The 71 SNPs $G_{1},\ldots,G_{71}$ are generated independently from a trinomial distribution in which the probabilities of $G_{j}$ being 0, 1, 2 are respectively equal to the proportions of $G_{j}$being 0, 1 and 2 in the UKHLS data (column 4,5,6 in Table S1) and the true causal effects of X-SNPs are from GIANT study (Table S1 last column) . The true value of the causal effect of X on Y is $\gamma_{X}=-0.2$. Outcomes Y are generated from

$Y=\gamma_{0}+\gamma_{X}X+\epsilon_{Y}$ (1)

where exposures X are generated from

$X=\beta_{0}+\sum_{j=1}^{J} \beta_{j}G_{j}+\epsilon_{X}$ (2)

To simulate data subject to unobserved confounding, the error terms in model (1) and (2) are respectively decomposed as $\epsilon_{Y}=U\gamma_{U}+\dot{\epsilon}_{Y}$ and $\epsilon_{X}=U+\dot{\epsilon}_{X}$, where $U$ is a zero-mean variable representing unobserved confounding, and $\dot{\varepsilon}_{Y}$ and $\dot{\epsilon}_{X}$ are not only mutually independent but jointly independent of $\left( Y,X,\mathbf{G}^{'},U \right)$. Parameter $\gamma_{U}=1$ indexes the extent of unobserved confounding by controlling the strength and sign of the correlation between $\epsilon_{Y}$ and $\epsilon_{X}$, such that there is no unobserved confounding if $\gamma_{U}=0$. $U, \dot{\epsilon}_{X}$and$\dot{\epsilon}_{Y}$ all independently generated from standard normal distributions.

S1.2 Some SNPs are invalid

The outcomes Y and X are respectively generated from

$Y=\gamma_{0}+\gamma_{X}X+\sum_{j=1}^{J} \left( \alpha_{j}+\theta_{j} \right)G_{j}+\epsilon_{Y}$ (3)

$$X=\beta_{0}+\sum_{j=1}^{J} {(\beta}_{j}+\theta_{j})G_{j}+\epsilon_{X}\equiv b_{0}+\sum_{j=1}^{J} b_{j}G_{j}+\epsilon_{X} (4)$$

where $\theta_{j}=0$ if this SNP is a valid IV. The three different pleiotropy scenarios are defined as follows:

1. Direct pleiotropy under which the InSIDE condition holds so that $\theta_{j}=0$ for all $j=1,\ldots,J$, where the direct pleiotropy is balanced by generating $\sum_{s=1}^{S} \alpha_{s}=0$, $\alpha_{s}\sim U\left( -0.2,0.2 \right)$, $s=1,\ldots,S$, where $S$ is the number of invalid SNPs. $U(a,b)$ indicates the continuous uniform distribution on real interval $(a,b)$. In this scenario, individual SNPs lead to direct pleiotropy but the full set of SNPs does not. The true SNP-exposure association equals the causal effect of the SNP: $b_{j}=\beta_{j}$.
2. As in Scenario 1 (including that $\theta_{j}=0$ for all $j=1,\ldots,J,$ and$b_{j}=\beta_{j}$) but the direct pleiotropy is now unbalanced with $\sum_{s=1}^{S} \alpha_{s}>0$and$\alpha_{s}\sim U\left( 0,0.2 \right), s=1,\ldots,S$.
3. As in Scenario 2, except there is indirect pleiotropy with positive$\theta_{s}\sim U\left( 0,0.4 \right), s=1,\ldots,S$ and InSIDE fails because the true association between SNP $G_{s}$ and exposure is $b_{s}=\beta_{s}+\theta_{s}$.

The error terms of (3) and (4) are generated the same way as above. For each of these scenarios, the performance of each estimator is assessed for $S=10, 20 and 30$ invalid IVs corresponds respectively to 14%, 28% and 42% of the SNPs; these invalid SNPs are randomly selected from 71 SNPs at each simulation; these scenarios all satisfy the requirement that less than 50% of the SNPs are invalid.

Table S1 The effect/Non-effect Allele, frequency of 0/1/2 and Beta and standard errors from GIANT study for 71 common BMI-associated SNPs

| Genetic variant | Effect Allele | Non-effect allele | Frequency of Zero | Frequency of One | Frequency of Two | EAF of UKHLS | EAF of GIANT | Beta (se) of GIANT |
| --- | --- | --- | --- | --- | --- | --- | --- | --- |
| rs1558902 | A | T | 0.357 | 0.477 | 0.165 | 0.404 | 0.415 | 0.082 (0.003) |
| rs10938397 | G | A | 0.313 | 0.489 | 0.198 | 0.442 | 0.434 | 0.040 (0.003) |
| rs10182181 | G | A | 0.259 | 0.499 | 0.242 | 0.491 | 0.462 | 0.031 (0.003) |
| rs7138803 | A | G | 0.399 | 0.465 | 0.136 | 0.369 | 0.384 | 0.032 (0.003) |
| rs3101336 | C | T | 0.156 | 0.480 | 0.363 | 0.604 | 0.613 | 0.033 (0.003) |
| rs9540493 | A | G | 0.301 | 0.497 | 0.202 | 0.451 | 0.452 | 0.021 (0.003) |
| rs11030104 | A | G | 0.041 | 0.321 | 0.638 | 0.499 | 0.792 | 0.041 (0.004) |
| rs12566985 | G | A | 0.319 | 0.490 | 0.191 | 0.436 | 0.446 | 0.024 (0.003) |
| rs13021737 | G | A | 0.027 | 0.273 | 0.700 | 0.836 | 0.828 | 0.060 (0.004) |
| rs17405819 | T | C | 0.087 | 0.414 | 0.499 | 0.706 | 0.700 | 0.022 (0.003) |
| rs1516725 | C | T | 0.019 | 0.232 | 0.749 | 0.865 | 0.872 | 0.045 (0.005) |
| rs12016871 | T | C | 0.676 | 0.296 | 0.028 | 0.176 | 0.203 | 0.030 (0.005) |
| rs2650492 | A | G | 0.498 | 0.411 | 0.091 | 0.296 | 0.303 | 0.021 (0.004) |
| rs12885454 | C | A | 0.130 | 0.459 | 0.411 | 0.640 | 0.642 | 0.021 (0.003) |
| rs1928295 | T | C | 0.190 | 0.485 | 0.325 | 0.567 | 0.548 | 0.019 (0.003) |
| rs11057405 | G | A | 0.011 | 0.196 | 0.793 | 0.891 | 0.901 | 0.031 (0.006) |
| rs12940622 | G | A | 0.191 | 0.499 | 0.309 | 0.559 | 0.575 | 0.018 (0.003) |
| rs16951275 | T | C | 0.051 | 0.350 | 0.599 | 0.774 | 0.784 | 0.031 (0.004) |
| rs12446632 | G | A | 0.022 | 0.240 | 0.738 | 0.858 | 0.865 | 0.040 (0.005) |
| rs4740619 | T | C | 0.206 | 0.493 | 0.300 | 0.547 | 0.542 | 0.018 (0.003) |
| rs10968576 | G | A | 0.460 | 0.437 | 0.103 | 0.321 | 0.320 | 0.025 (0.003) |
| rs3810291 | A | G | 0.109 | 0.437 | 0.454 | 0.672 | 0.666 | 0.028 (0.004) |
| rs657452 | A | G | 0.376 | 0.467 | 0.157 | 0.391 | 0.394 | 0.023 (0.003) |
| rs17724992 | A | G | 0.068 | 0.388 | 0.543 | 0.737 | 0.746 | 0.019 (0.004) |
| rs2176598 | T | C | 0.562 | 0.374 | 0.064 | 0.251 | 0.251 | 0.020 (0.004) |
| rs12401738 | A | G | 0.384 | 0.472 | 0.144 | 0.380 | 0.352 | 0.021 (0.003) |
| rs11847697 | T | C | 0.915 | 0.083 | 0.002 | 0.043 | 0.042 | 0.049 (0.008) |
| rs2112347 | T | G | 0.132 | 0.460 | 0.408 | 0.638 | 0.629 | 0.026 (0.003) |
| rs17001654 | G | C | 0.720 | 0.258 | 0.022 | 0.151 | 0.153 | 0.031 (0.005) |
| rs7141420 | T | C | 0.241 | 0.504 | 0.255 | 0.508 | 0.527 | 0.024 (0.003) |
| rs1000940 | G | A | 0.481 | 0.427 | 0.092 | 0.306 | 0.320 | 0.019 (0.003) |
| rs6567160 | C | T | 0.580 | 0.361 | 0.059 | 0.240 | 0.236 | 0.056 (0.004) |
| rs11583200 | C | T | 0.382 | 0.466 | 0.152 | 0.385 | 0.396 | 0.018 (0.003) |
| rs7903146 | C | T | 0.083 | 0.410 | 0.507 | 0.712 | 0.713 | 0.023 (0.003) |
| rs2365389 | C | T | 0.170 | 0.472 | 0.358 | 0.594 | 0.582 | 0.020 (0.003) |
| rs1808579 | C | T | 0.235 | 0.501 | 0.264 | 0.514 | 0.534 | 0.017 (0.003) |
| rs6804842 | G | A | 0.178 | 0.489 | 0.333 | 0.578 | 0.575 | 0.019 (0.003) |
| rs6477694 | C | T | 0.425 | 0.457 | 0.118 | 0.346 | 0.365 | 0.017 (0.003) |
| rs9400239 | C | T | 0.084 | 0.420 | 0.496 | 0.706 | 0.688 | 0.019 (0.003) |
| rs3817334 | T | C | 0.343 | 0.489 | 0.167 | 0.412 | 0.407 | 0.026 (0.003) |
| rs2075650 | A | G | 0.024 | 0.248 | 0.728 | 0.852 | 0.848 | 0.026 (0.005) |
| rs2207139 | G | A | 0.693 | 0.279 | 0.028 | 0.167 | 0.177 | 0.045 (0.004) |
| rs1016287 | T | C | 0.501 | 0.406 | 0.093 | 0.296 | 0.287 | 0.023 (0.003) |
| rs543874 | G | A | 0.628 | 0.330 | 0.042 | 0.207 | 0.193 | 0.048 (0.004) |
| rs9925964 | A | G | 0.123 | 0.460 | 0.416 | 0.646 | 0.620 | 0.019 (0.003) |
| rs758747 | T | C | 0.514 | 0.404 | 0.082 | 0.284 | 0.265 | 0.023 (0.004) |
| rs2033529 | G | A | 0.512 | 0.407 | 0.081 | 0.285 | 0.293 | 0.019 (0.003) |
| rs13107325 | T | C | 0.847 | 0.146 | 0.007 | 0.080 | 0.072 | 0.048 (0.007) |
| rs7599312 | G | A | 0.073 | 0.393 | 0.534 | 0.730 | 0.724 | 0.022 (0.003) |
| rs2121279 | T | C | 0.764 | 0.220 | 0.016 | 0.126 | 0.152 | 0.025 (0.004) |
| rs9374842 | T | C | 0.055 | 0.349 | 0.596 | 0.771 | 0.744 | 0.023 (0.004) |
| rs4787491 | G | A | 0.215 | 0.497 | 0.288 | 0.536 | 0.510 | 0.022 (0.004) |
| rs13191362 | A | G | 0.015 | 0.214 | 0.771 | 0.878 | 0.879 | 0.028 (0.005) |
| rs2820292 | C | A | 0.187 | 0.487 | 0.326 | 0.569 | 0.555 | 0.020 (0.003) |
| rs7899106 | G | A | 0.899 | 0.098 | 0.003 | 0.052 | 0.052 | 0.040 (0.007) |
| rs11165643 | T | C | 0.168 | 0.481 | 0.351 | 0.592 | 0.583 | 0.022 (0.003) |
| rs4256980 | G | C | 0.116 | 0.449 | 0.435 | 0.659 | 0.646 | 0.021 (0.003) |
| rs13078960 | G | T | 0.633 | 0.323 | 0.044 | 0.206 | 0.196 | 0.030 (0.004) |
| rs11126666 | A | G | 0.555 | 0.379 | 0.066 | 0.255 | 0.283 | 0.021 (0.003) |
| rs7243357 | T | G | 0.028 | 0.279 | 0.692 | 0.832 | 0.812 | 0.022 (0.004) |
| rs2287019 | C | T | 0.035 | 0.298 | 0.667 | 0.816 | 0.804 | 0.036 (0.004) |
| rs1167827 | G | A | 0.186 | 0.487 | 0.327 | 0.570 | 0.553 | 0.020 (0.003) |
| rs16851483 | T | G | 0.871 | 0.125 | 0.004 | 0.067 | 0.066 | 0.048 (0.008) |
| rs29941 | G | A | 0.109 | 0.438 | 0.453 | 0.672 | 0.669 | 0.018 (0.003) |
| rs9641123 | C | G | 0.353 | 0.487 | 0.159 | 0.403 | 0.430 | 0.029 (0.005) |
| rs3736485 | A | G | 0.294 | 0.496 | 0.210 | 0.458 | 0.454 | 0.018 (0.003) |
| rs3849570 | A | C | 0.430 | 0.451 | 0.119 | 0.345 | 0.359 | 0.019 (0.003) |
| rs1528435 | T | C | 0.147 | 0.463 | 0.390 | 0.622 | 0.631 | 0.018 (0.003) |
| rs2033732 | C | T | 0.063 | 0.378 | 0.558 | 0.748 | 0.747 | 0.019 (0.004) |
| rs12429545 | A | G | 0.753 | 0.230 | 0.017 | 0.132 | 0.133 | 0.033 (0.005) |
| rs12286929 | G | A | 0.223 | 0.496 | 0.281 | 0.529 | 0.523 | 0.022 (0.003) |

Table S2 Simulation results for multiple instruments (All SNPs are valid)

| Methods | Median | Mean (SD) | Mean SE | MSE | Coverage % | Power % |
| --- | --- | --- | --- | --- | --- | --- |
| True value | **-0.2** |  |  |  |  |  |
| One-sample strategy |  |  |  |  |  |  |
| SPRS | -0.196 | -0.202 (0.104) | 0.104 | 0.011 | 96.0 | 49.2 |
| IPRS | -0.009 | -0.008 (0.068) | 0.068 | 0.041 | 21.7 | 4.3 |
| 2SLS | -0.009 | -0.007 (0.067) | 0.068 | 0.042 | 20.7 | 4.3 |
| LIML | -0.200 | -0.206 (0.121) | 0.096 | 0.015 | 89.0 | 57.9 |
| Weighted Egger | 0.057 | 0.054 (0.119) | 0.122 | 0.079 | 43.7 | 7.3 |
| Weighted Median | 0.005 | 0.003 (0.090) | 0.104 | 0.049 | 49.5 | 2.6 |
| sisVIVE - SPRS | -0.196 | -0.202 (0.104) | 0.104 | 0.011 | 95.9 | 48.8 |
| sisVIVE - IPRS | -0.009 | -0.008 (0.068) | 0.071 | 0.042 | 23.4 | 3.8 |
| Two-sample strategy  True precision ${\tilde{\boldsymbol{b}}}_{\boldsymbol{j}}\boldsymbol{=}\boldsymbol{\beta}_{\boldsymbol{j}}$ | | | |  |  |  |
| EPRS | -0.200 | -0.203 (0.095) | 0.095 | 0.009 | 95.8 | 58.6 |
| 2SLS | -0.201 | -0.199 (0.085) | 0.089 | 0.007 | 95.8 | 61.3 |
| Weighted Egger | -0.195 | -0.208 (0.218) | 0.220 | 0.047 | 95.5 | 16.5 |
| Weighted Median | -0.202 | -0.200 (0.126) | 0.142 | 0.016 | 97.4 | 26.2 |
| sisVIVE - SPRS | -0.196 | -0.202 (0.104) | 0.104 | 0.011 | 96.0 | 49.2 |
| sisVIVE - 2SLS | -0.201 | -0.199 (0.085) | 0.089 | 0.007 | 95.8 | 61.3 |
| Precise: ${\tilde{\boldsymbol{b}}}_{\boldsymbol{j}}\boldsymbol{\sim N(}\boldsymbol{\beta}_{\boldsymbol{j}}\boldsymbol{,}\boldsymbol{0.01}^{\boldsymbol{2}}\boldsymbol{)}$ | |  |  |  |  |  |
| EPRS | -0.202 | -0.204 (0.100) | 0.100 | 0.010 | 95.5 | 53.7 |
| 2SLS | -0.181 | -0.178 (0.081) | 0.084 | 0.007 | 95.2 | 57.5 |
| Weighted Egger | -0.128 | -0.134 (0.175) | 0.171 | 0.034 | 93.5 | 14.0 |
| Weighted Median | -0.177 | -0.176 (0.114) | 0.130 | 0.014 | 97.6 | 8.4 |
| sisVIVE - SPRS | -0.196 | -0.202 (0.104) | 0.104 | 0.011 | 96.0 | 49.2 |
| sisVIVE - 2SLS | -0.181 | -0.178 (0.081) | 0.084 | 0.007 | 95.2 | 57.5 |
| Imprecise: ${\tilde{\boldsymbol{b}}}_{\boldsymbol{j}}\boldsymbol{\sim N(}\boldsymbol{\beta}_{\boldsymbol{j}}\boldsymbol{,}\boldsymbol{0.05}^{\boldsymbol{2}}\boldsymbol{)}$ | | |  |  |  |  |
| EPRS | -0.189 | -0.218 (0.215) | 0.210 | 0.041 | 96.4 | 9.7 |
| 2SLS | -0.049 | -0.050 (0.044) | 0.045 | 0.024 | 9.9 | 19.4 |
| Weighted Egger | -0.046 | -0.047 (0.072) | 0.076 | 0.029 | 46.0 | 8.7 |
| Weighted Median | -0.048 | -0.047 (0.058) | 0.066 | 0.027 | 33.1 | 4.5 |
| sisVIVE - SPRS | -0.196 | -0.202 (0.104) | 0.104 | 0.011 | 96.0 | 49.1 |
| sisVIVE - 2SLS | -0.049 | -0.050 (0.044) | 0.045 | 0.024 | 9.9 | 19.4 |

Table S3 Simulation results for multiple instruments (InSIDE holds, 10 SNPs have Balance pleiotropy with$\alpha_{s}\sim U\left( -0.2,0.2 \right))$

| Methods | Median | Mean (SD) | Mean SE | MSE | Coverage % | Power % |
| --- | --- | --- | --- | --- | --- | --- |
| True value | **-0.2** |  |  |  |  |  |
| One-sample strategy |  |  |  |  |  |  |
| SPRS | -0.192 | -0.200 (0.231) | 0.107 | 0.053 | 64.2 | 52.9 |
| IPRS | 0.000 | -0.008 (0.157) | 0.157 | 0.061 | 75.7 | 4.1 |
| Weighted Egger | 0.064 | 0.059 (0.276) | 0.268 | 0.143 | 82.0 | 5.4 |
| Weighted Median | -0.004 | -0.002 (0.108) | 0.113 | 0.051 | 59.4 | 3.3 |
| sisVIVE - SPRS | -0.165 | -0.169 (0.117) | 0.110 | 0.015 | 91.6 | 31.0 |
| sisVIVE - IPRS | -0.008 | -0.007 (0.083) | 0.077 | 0.044 | 31.3 | 7.3 |
| Two-sample strategy |  |  |  |  |  |  |
| True: ${\tilde{\boldsymbol{b}}}_{\boldsymbol{j}}\boldsymbol{=}\boldsymbol{\beta}_{\boldsymbol{j}}$ |  |  |  |  |  |  |
| EPRS | -0.189 | -0.195 (0.203) | 0.097 | 0.044 | 63.2 | 54.7 |
| 2SLS | -0.189 | -0.195 (0.203) | 0.197 | 0.041 | 94.1 | 18.5 |
| Weighted Egger | -0.201 | -0.198 (0.542) | 0.490 | 0.293 | 91.3 | 24.6 |
| Weighted Median | -0.199 | -0.203 (0.152) | 0.155 | 0.023 | 95.9 | 25.4 |
| sisVIVE - SPRS | -0.210 | -0.210 (0.120) | 0.113 | 0.015 | 94.3 | 46.8 |
| sisVIVE - 2SLS | -0.209 | -0.204 (0.105) | 0.096 | 0.011 | 92.2 | 57.9 |
| Precise: ${\tilde{\boldsymbol{b}}}_{\boldsymbol{j}}\boldsymbol{\sim N(}\boldsymbol{\beta}_{\boldsymbol{j}}\boldsymbol{,}\boldsymbol{0.01}^{\boldsymbol{2}}\boldsymbol{)}$ | |  |  |  |  |  |
| EPRS | -0.180 | -0.197 (0.219) | 0.103 | 0.048 | 64.9 | 51.1 |
| 2SLS | -0.163 | -0.173 (0.191) | 0.187 | 0.037 | 94.3 | 16.1 |
| Weighted Egger | -0.124 | -0.124 (0.411) | 0.380 | 0.175 | 91.7 | 8.4 |
| Weighted Median | -0.173 | -0.177 (0.137) | 0.142 | 0.019 | 96.1 | 22.3 |
| sisVIVE - SPRS | -0.200 | -0.204 (0.118) | 0.112 | 0.014 | 94.4 | 44.7 |
| sisVIVE - 2SLS | -0.179 | -0.182 (0.098) | 0.091 | 0.010 | 92.7 | 52.3 |
| Imprecise: ${\tilde{\boldsymbol{b}}}_{\boldsymbol{j}}\boldsymbol{\sim N(}\boldsymbol{\beta}_{\boldsymbol{j}}\boldsymbol{,}\boldsymbol{0.05}^{\boldsymbol{2}}\boldsymbol{)}$ | |  |  |  |  |  |
| EPRS | -0.195 | -0.208 (0.558) | 0.249 | 0.311 | 69.0 | 37.6 |
| 2SLS | -0.048 | -0.045 (0.101) | 0.100 | 0.034 | 65.4 | 8.1 |
| Weighted Egger | -0.046 | -0.040 (0.174) | 0.168 | 0.056 | 82.4 | 6.4 |
| Weighted Median | -0.049 | -0.050 (0.068) | 0.071 | 0.028 | 42.9 | 8.8 |
| sisVIVE - SPRS | -0.179 | -0.185 (0.110) | 0.111 | 0.012 | 94.8 | 35.4 |
| sisVIVE - 2SLS | -0.052 | -0.052 (0.055) | 0.048 | 0.025 | 17.3 | 22.6 |

Table S4 Simulation results for multiple instruments (InSIDE holds, 20 SNPs have Balance pleiotropy with$\alpha_{s}\sim U\left( -0.2,0.2 \right))$

| Methods | Median | Mean (SD) | Mean SE | MSE | Coverage% | Power% |
| --- | --- | --- | --- | --- | --- | --- |
| True value | **-0.2** |  |  |  |  |  |
| One-sample strategy |  |  |  |  |  |  |
| SPRS | -0.205 | -0.214 (0.304) | 0.110 | 0.092 | 52.6 | 60.3 |
| IPRS | -0.012 | -0.018 (0.218) | 0.212 | 0.081 | 84.9 | 5.2 |
| Weighted Egger | 0.023 | 0.025 (0.371) | 0.363 | 0.188 | 90.0 | 6.9 |
| Weighted Median | -0.001 | -0.004 (0.129) | 0.124 | 0.055 | 64.0 | 4.7 |
| sisVIVE - SPRS | -0.129 | -0.126 (0.129) | 0.122 | 0.022 | 86.8 | 15.8 |
| sisVIVE - IPRS | -0.006 | -0.006 (0.101) | 0.084 | 0.048 | 37.4 | 9.5 |
| Two-sample strategy |  |  |  |  |  |  |
| True: ${\tilde{\boldsymbol{b}}}_{\boldsymbol{j}}\boldsymbol{=}\boldsymbol{\beta}_{\boldsymbol{j}}$ |  |  |  |  |  |  |
| EPRS | -0.204 | -0.211 (0.276) | 0.100 | 0.075 | 54.5 | 59.2 |
| 2SLS | -0.204 | -0.207 (0.269) | 0.268 | 0.073 | 94.4 | 11.7 |
| Weighted Egger | -0.216 | -0.193 (0.658) | 0.666 | 0.432 | 93.9 | 7.6 |
| Weighted Median | -0.201 | -0.204 (0.186) | 0.170 | 0.034 | 94.3 | 23.2 |
| sisVIVE - SPRS | -0.204 | -0.210 (0.147) | 0.127 | 0.022 | 92.5 | 36.6 |
| sisVIVE - 2SLS | -0.200 | -0.201 (0.129) | 0.104 | 0.017 | 89.3 | 49.0 |
| Precise: ${\tilde{\boldsymbol{b}}}_{\boldsymbol{j}}\boldsymbol{\sim N(}\boldsymbol{\beta}_{\boldsymbol{j}}\boldsymbol{,}\boldsymbol{0.01}^{\boldsymbol{2}}\boldsymbol{)}$ | |  |  |  |  |  |
| EPRS | -0.195 | -0.214 (0.291) | 0.106 | 0.084 | 54.9 | 60.4 |
| 2SLS | -0.175 | -0.186 (0.255) | 0.263 | 0.065 | 94.2 | 11.4 |
| Weighted Egger | -0.119 | -0.138 (0.542) | 0.598 | 0.297 | 93.3 | 7.2 |
| Weighted Median | -0.167 | -0.175 (0.166) | 0.165 | 0.028 | 93.9 | 20.6 |
| sisVIVE - SPRS | -0.195 | -0.199 (0.138) | 0.126 | 0.019 | 93.1 | 33.6 |
| sisVIVE - 2SLS | -0.176 | -0.177 (0.119) | 0.099 | 0.015 | 89.0 | 44.8 |
| Imprecise: ${\tilde{\boldsymbol{b}}}_{\boldsymbol{j}}\boldsymbol{\sim N(}\boldsymbol{\beta}_{\boldsymbol{j}}\boldsymbol{,}\boldsymbol{0.05}^{\boldsymbol{2}}\boldsymbol{)}$ | |  |  |  |  |  |
| EPRS | -0.238 | -0.267 (0.670) | 0.260 | 0.453 | 56.1 | 59.3 |
| 2SLS | -0.062 | -0.059 (0.141) | 0.249 | 0.040 | 79.2 | 8.5 |
| Weighted Egger | -0.040 | -0.050 (0.250) | 0.476 | 0.085 | 86.9 | 7.8 |
| Weighted Median | -0.048 | -0.049 (0.085) | 0.152 | 0.015 | 47.6 | 11.3 |
| sisVIVE - SPRS | -0.151 | -0.153 (0.115) | 0.123 | 0.027 | 92.8 | 17.1 |
| sisVIVE - 2SLS | -0.049 | -0.052 (0.067) | 0.053 | 0.026 | 25.2 | 22.8 |

Table S5 Simulation results for multiple instruments (InSIDE holds, 30 SNPs have Balance pleiotropy with$\alpha_{s}\sim U\left( -0.2,0.2 \right))$

| Methods | Median | Mean (SD) | Mean SE | MSE | Coverage % | Power % |
| --- | --- | --- | --- | --- | --- | --- |
| True value | **-0.2** |  |  |  |  |  |
| One-sample strategy |  |  |  |  |  |  |
| SPRS | -0.190 | -0.194 (0.376) | 0.113 | 0.141 | 41.8 | 63.6 |
| IPRS | -0.014 | -0.010 (0.269) | 0.257 | 0.108 | 86.7 | 6.4 |
| Weighted Egger | 0.065 | 0.053 (0.450) | 0.440 | 0.266 | 90.9 | 5.3 |
| Weighted Median | -0.008 | -0.005 (0.165) | 0.139 | 0.065 | 65.3 | 7.8 |
| sisVIVE - SPRS | -0.091 | -0.095 (0.157) | 0.140 | 0.036 | 83.5 | 11.7 |
| sisVIVE - IPRS | -0.008 | -0.008 (0.132) | 0.097 | 0.054 | 49.2 | 15.1 |
| Two-sample strategy |  |  |  |  |  |  |
| True: ${\tilde{\boldsymbol{b}}}_{\boldsymbol{j}}\boldsymbol{=}\boldsymbol{\beta}_{\boldsymbol{j}}$ |  |  |  |  |  |  |
| EPRS | -0.192 | -0.194 (0.334) | 0.102 | 0.111 | 45.2 | 63.4 |
| 2SLS | -0.190 | -0.189 (0.326) | 0.323 | 0.106 | 94.5 | 9.0 |
| Weighted Egger | -0.203 | -0.193 (0.837) | 0.801 | 0.670 | 92.7 | 7.6 |
| Weighted Median | -0.200 | -0.202 (0.232) | 0.191 | 0.054 | 91.8 | 22.4 |
| sisVIVE - SPRS | -0.204 | -0.213 (0.188) | 0.149 | 0.036 | 90.9 | 30.7 |
| sisVIVE - 2SLS | -0.206 | -0.201 (0.164) | 0.120 | 0.027 | 86.1 | 42.7 |
| Precise: ${\tilde{\boldsymbol{b}}}_{\boldsymbol{j}}\boldsymbol{\sim N(}\boldsymbol{\beta}_{\boldsymbol{j}}\boldsymbol{,}\boldsymbol{0.01}^{\boldsymbol{2}}\boldsymbol{)}$ | |  |  |  |  |  |
| EPRS | -0.195 | -0.198 (0.356) | 0.109 | 0.127 | 45.3 | 63.5 |
| 2SLS | -0.173 | -0.173 (0.311) | 0.305 | 0.097 | 94.7 | 9.7 |
| Weighted Egger | -0.157 | -0.125 (0.666) | 0.619 | 0.448 | 91.8 | 7.1 |
| Weighted Median | -0.183 | -0.179 (0.217) | 0.176 | 0.048 | 91.2 | 21.6 |
| sisVIVE - SPRS | -0.192 | -0.202 (0.179) | 0.147 | 0.032 | 90.4 | 28.4 |
| sisVIVE - 2SLS | -0.187 | -0.181 (0.157) | 0.114 | 0.025 | 85.4 | 40.2 |
| Imprecise: ${\tilde{\boldsymbol{b}}}_{\boldsymbol{j}}\boldsymbol{\sim N(}\boldsymbol{\beta}_{\boldsymbol{j}}\boldsymbol{,}\boldsymbol{0.05}^{\boldsymbol{2}}\boldsymbol{)}$ | |  |  |  |  |  |
| EPRS | -0.219 | -0.239 (0.820) | 0.272 | 0.672 | 47.6 | 57.1 |
| 2SLS | -0.052 | -0.057 (0.174) | 0.163 | 0.051 | 83.2 | 9.0 |
| Weighted Egger | -0.085 | -0.074 (0.271) | 0.274 | 0.089 | 93.1 | 7.0 |
| Weighted Median | -0.053 | -0.056 (0.107) | 0.085 | 0.032 | 56.4 | 19.3 |
| sisVIVE - SPRS | -0.122 | -0.131 (0.134) | 0.140 | 0.023 | 91.6 | 9.6 |
| sisVIVE - 2SLS | -0.052 | -0.055 (0.084) | 0.061 | 0.028 | 35.6 | 23.3 |

Table S6 Simulation results for multiple instruments (InSIDE holds, 10 have positive pleiotropy with$\alpha_{s}\sim U\left( 0,0.2 \right))$

| Methods | Median | Mean (SD) | Mean SE | MSE | Coverage % | Power % |
| --- | --- | --- | --- | --- | --- | --- |
| True value | **-0.2** |  |  |  |  |  |
| One-sample strategy |  |  |  |  |  |  |
| SPRS | 0.341 | 0.342 (0.145) | 0.092 | 0.315 | 9.0 | 86.4 |
| IPRS | 0.271 | 0.270 (0.124) | 0.150 | 0.236 | 7.0 | 42.5 |
| Weighted Egger | 0.190 | 0.199 (0.258) | 0.257 | 0.225 | 64.8 | 13.3 |
| Weighted Median | 0.073 | 0.076 (0.110) | 0.114 | 0.088 | 30.6 | 9.2 |
| sisVIVE - SPRS | -0.104 | -0.106 (0.115) | 0.106 | 0.022 | 81.2 | 15.1 |
| sisVIVE - IPRS | 0.041 | 0.042 (0.083) | 0.078 | 0.065 | 14.5 | 11.6 |
| Two-sample strategy |  |  |  |  |  |  |
| True: ${\tilde{\boldsymbol{b}}}_{\boldsymbol{j}}\boldsymbol{=}\boldsymbol{\beta}_{\boldsymbol{j}}$ |  |  |  |  |  |  |
| EPRS | 0.248 | 0.251 (0.136) | 0.084 | 0.222 | 2.0 | 75.4 |
| 2SLS | 0.245 | 0.255 (0.139) | 0.192 | 0.226 | 24.7 | 15.3 |
| Weighted Egger | -0.275 | -0.214 (0.483) | 0.473 | 0.233 | 93.7 | 6.4 |
| Weighted Median | -0.103 | -0.099 (0.154) | 0.152 | 0.034 | 90.3 | 10.6 |
| sisVIVE - SPRS | -0.126 | -0.130 (0.118) | 0.108 | 0.019 | 85.3 | 20.0 |
| sisVIVE - 2SLS | -0.131 | -0.128 (0.109) | 0.096 | 0.017 | 84.2 | 29.5 |
| Precise: ${\tilde{\boldsymbol{b}}}_{\boldsymbol{j}}\boldsymbol{\sim N(}\boldsymbol{\beta}_{\boldsymbol{j}}\boldsymbol{,}\boldsymbol{0.01}^{\boldsymbol{2}}\boldsymbol{)}$ | |  |  |  |  |  |
| EPRS | 0.232 | 0.246 (0.148) | 0.089 | 0.221 | 3.4 | 67.4 |
| 2SLS | 0.210 | 0.223 (0.136) | 0.182 | 0.197 | 30.6 | 14.1 |
| Weighted Egger | -0.087 | -0.083 (0.138) | 0.367 | 0.144 | 92.6 | 5.6 |
| Weighted Median | -0.096 | -0.091 (0.147) | 0.140 | 0.033 | 87.9 | 8.7 |
| sisVIVE - SPRS | -0.128 | -0.130 (0.116) | 0.108 | 0.018 | 85.6 | 20.5 |
| sisVIVE - 2SLS | -0.116 | -0.115 (0.104) | 0.091 | 0.018 | 81.7 | 28.7 |
| Imprecise: ${\tilde{\boldsymbol{b}}}_{\boldsymbol{j}}\boldsymbol{\sim N(}\boldsymbol{\beta}_{\boldsymbol{j}}\boldsymbol{,}\boldsymbol{0.05}^{\boldsymbol{2}}\boldsymbol{)}$ | |  |  |  |  |  |
| EPRS | 0.235 | 0.248 (0.441) | 0.199 | 0.394 | 35.6 | 44.0 |
| 2SLS | 0.062 | 0.065 (0.099) | 0.097 | 0.080 | 21.9 | 10.3 |
| Weighted Egger | 0.054 | 0.057 (0.169) | 0.164 | 0.094 | 64.0 | 7.3 |
| Weighted Median | -0.017 | -0.016 (0.070) | 0.071 | 0.039 | 25.2 | 4.6 |
| sisVIVE - SPRS | -0.126 | -0.129 (0.109) | 0.107 | 0.017 | 87.0 | 17.8 |
| sisVIVE - 2SLS | -0.030 | -0.030 (0.056) | 0.049 | 0.032 | 8.1 | 13.4 |

Table S7 Simulation results for multiple instruments (InSIDE holds, 20 SNPs have positive pleiotropy with$\alpha_{s}\sim U\left( 0,0.2 \right)$)

| Methods | Median | Mean (SD) | Mean SE | MSE | Coverage % | Power % |
| --- | --- | --- | --- | --- | --- | --- |
| True value | **-0.2** |  |  |  |  |  |
| One-sample strategy |  |  |  |  |  |  |
| SPRS | 0.869 | 0.880 (0.190) | 0.110 | 1.203 | 0 | 100 |
| IPRS | 0.550 | 0.554 (0.162) | 0.194 | 0.594 | 1 | 87.4 |
| Weighted Egger | 0.343 | 0.350 (0.334) | 0.332 | 0.414 | 63.0 | 18.5 |
| Weighted Median | 0.172 | 0.181 (0.140) | 0.131 | 0.165 | 15.0 | 27.5 |
| sisVIVE - SPRS | 0.011 | 0.018 (0.136) | 0.121 | 0.066 | 54.0 | 8.1 |
| sisVIVE - IPRS | 0.123 | 0.128 (0.114) | 0.087 | 0.121 | 6.0 | 35.1 |
| Two-sample strategy |  |  |  |  |  |  |
| True: ${\tilde{\boldsymbol{b}}}_{\boldsymbol{j}}\boldsymbol{=}\boldsymbol{\beta}_{\boldsymbol{j}}$ |  |  |  |  |  |  |
| EPRS | 0.686 | 0.701 (0.173) | 0.093 | 0.842 | 0 | 100 |
| 2SLS | 0.686 | 0.704 (0.171) | 0.247 | 0.846 | 0 | 92.3 |
| Weighted Egger | -0.300 | -0.222 (0.612) | 0.602 | 0.374 | 94.4 | 5.8 |
| Weighted Median | 0.031 | 0.052 (0.205) | 0.171 | 0.106 | 71.3 | 9.7 |
| sisVIVE - SPRS | 0.007 | 0.009 (0.136) | 0.117 | 0.062 | 53.2 | 8.7 |
| sisVIVE - 2SLS | 0.012 | 0.018 (0.138) | 0.011 | 0.067 | 49.9 | 11.5 |
| Precise: ${\tilde{\boldsymbol{b}}}_{\boldsymbol{j}}\boldsymbol{\sim N(}\boldsymbol{\beta}_{\boldsymbol{j}}\boldsymbol{,}\boldsymbol{0.01}^{\boldsymbol{2}}\boldsymbol{)}$ | |  |  |  |  |  |
| EPRS | 0.678 | 0.696 (0.198) | 0.099 | 0.842 | 0 | 99.9 |
| 2SLS | 0.613 | 0.625 (0.175) | 0.129 | 0.711 | 0.8 | 84.1 |
| Weighted Egger | -0.099 | -0.078 (0.485) | 0.469 | 0.250 | 93.7 | 6.0 |
| Weighted Median | 0.036 | 0.049 (0.184) | 0.160 | 0.096 | 66.2 | 7.0 |
| sisVIVE - SPRS | 0.003 | -0.001 (0.134) | 0.117 | 0.058 | 56.0 | 7.8 |
| sisVIVE - 2SLS | 0.009 | 0.016 (0.134) | 0.104 | 0.064 | 47.1 | 11.7 |
| Imprecise: ${\tilde{\boldsymbol{b}}}_{\boldsymbol{j}}\boldsymbol{\sim N(}\boldsymbol{\beta}_{\boldsymbol{j}}\boldsymbol{,}\boldsymbol{0.05}^{\boldsymbol{2}}\boldsymbol{)}$ | |  |  |  |  |  |
| EPRS | 0.672 | 0.712 (0.055) | 0.227 | 1.134 | 13.0 | 79.0 |
| 2SLS | 0.173 | 0.174 (0.118) | 0.072 | 0.154 | 15.5 | 24.2 |
| Weighted Egger | 0.131 | 0.135 (0.223) | 0.218 | 0.162 | 64.7 | 9.2 |
| Weighted Median | 0.022 | 0.025 (0.090) | 0.078 | 0.059 | 19.2 | 6.9 |
| sisVIVE - SPRS | -0.067 | -0.066 (0.113) | 0.118 | 0.031 | 74.6 | 4.2 |
| sisVIVE - 2SLS | -0.003 | -0.000 (0.069) | 0.053 | 0.045 | 7.4 | 13.2 |

Table S8 Simulation results for multiple instruments (InSIDE holds, 30 SNPs have positive pleiotropy with$\alpha_{s}\sim U\left( 0,0.2 \right)$)

| Methods | Median | Mean (SD) | Mean SE | MSE | Coverage % | Power % |
| --- | --- | --- | --- | --- | --- | --- |
| True value | **-0.2** |  |  |  |  |  |
| One-sample strategy |  |  |  |  |  |  |
| SPRS | 1.409 | 1.420 (0.242) | 0.151 | 2.683 | 0 | 100 |
| IPRS | 0.829 | 0.829 (0.184) | 0.225 | 1.092 | 0 | 99.1 |
| Weighted Egger | 0.514 | 0.524 (0.378) | 0.382 | 0.667 | 52.7 | 27.0 |
| Weighted Median | 0.316 | 0.344 (0.208) | 0.157 | 0.339 | 5.8 | 55.8 |
| sisVIVE - SPRS | 0.207 | 0.214 (0.178) | 0.157 | 0.203 | 23.9 | 28.7 |
| sisVIVE - IPRS | 0.283 | 0.297 (0.161) | 0.112 | 0.272 | 2.3 | 70.5 |
| Two-sample strategy |  |  |  |  |  |  |
| True: ${\tilde{\boldsymbol{b}}}_{\boldsymbol{j}}\boldsymbol{=}\boldsymbol{\beta}_{\boldsymbol{j}}$ |  |  |  |  |  |  |
| EPRS | 1.144 | 1.154 (0.209) | 0.119 | 1.880 | 0 | 100 |
| 2SLS | 1.142 | 1.148 (0.194) | 0.280 | 1.854 | 0 | 100 |
| Weighted Egger | -0.222 | -0.197 (0.702) | 0.674 | 0.493 | 94.2 | 6.6 |
| Weighted Median | 0.247 | 0.311 (0.328) | 0.206 | 0.363 | 38.2 | 31.1 |
| sisVIVE - SPRS | 0.248 | 0.252 (0.168) | 0.135 | 0.233 | 13.0 | 47.8 |
| sisVIVE - 2SLS | 0.276 | 0.288 (0.192) | 0.140 | 0.274 | 10.9 | 50.1 |
| Precise: ${\tilde{\boldsymbol{b}}}_{\boldsymbol{j}}\boldsymbol{\sim N(}\boldsymbol{\beta}_{\boldsymbol{j}}\boldsymbol{,}\boldsymbol{0.01}^{\boldsymbol{2}}\boldsymbol{)}$ | |  |  |  |  |  |
| EPRS | 1.141 | 1.158 (0.239) | 0.126 | 1.901 | 0 | 100 |
| 2SLS | 1.019 | 1.028 (0.203) | 0.268 | 1.549 | 0 | 99.9 |
| Weighted Egger | 0.045 | 0.010 (0.568) | 0.527 | 0.366 | 91.1 | 6.8 |
| Weighted Median | 0.246 | 0.287 (0.290) | 0.193 | 0.321 | 35.2 | 30.7 |
| sisVIVE - SPRS | 0.219 | 0.215 (0.164) | 0.134 | 0.199 | 17.5 | 40.2 |
| sisVIVE - 2SLS | 0.255 | 0.262 (0.192) | 0.133 | 0.250 | 12.4 | 49.1 |
| Imprecise: ${\tilde{\boldsymbol{b}}}_{\boldsymbol{j}}\boldsymbol{\sim N(}\boldsymbol{\beta}_{\boldsymbol{j}}\boldsymbol{,}\boldsymbol{0.05}^{\boldsymbol{2}}\boldsymbol{)}$ | |  |  |  |  |  |
| EPRS | 1.160 | 1.184 (0.631) | 0.283 | 2.313 | 4.4 | 92.8 |
| 2SLS | 0.284 | 0.287 (0.143) | 0.152 | 0.258 | 10.1 | 48.5 |
| Weighted Egger | 0.251 | 0.250 (0.253) | 0.257 | 0.266 | 57.6 | 16.1 |
| Weighted Median | 0.076 | 0.092 (0.121) | 0.088 | 0.100 | 9.5 | 18.6 |
| sisVIVE - SPRS | 0.012 | 0.015 (0.129) | 0.134 | 0.063 | 61.7 | 5.1 |
| sisVIVE - 2SLS | 0.054 | 0.063 (0.096) | 0.063 | 0.078 | 4.4 | 24.9 |

Table S9 Simulation results for multiple instruments (10 SNPs have positive direct and indirect pleiotropy with$\alpha_{s}\sim U\left( 0,0.2 \right), \theta_{s}\sim U(0, 0.4))$

| Methods | Median | Mean (SD) | Mean SE | MSE | Coverage % | Power % |
| --- | --- | --- | --- | --- | --- | --- |
| True value | **-0.2** |  |  |  |  |  |
| One-sample strategy |  |  |  |  |  |  |
| SPRS | 0.578 | 0.574 (0.079) | 0.047 | 0.606 | 0 | 100 |
| IPRS | 0.937 | 0.938 (0.078) | 0.056 | 1.301 | 0 | 100 |
| Weighted Egger | 1.090 | 1.093 (0.097) | 0.061 | 1.682 | 0 | 100 |
| Weighted Median | 1.000 | 0.998 (0.115) | 0.065 | 1.449 | 0 | 100 |
| sisVIVE - SPRS | 0.527 | 0.517 (0.145) | 0.115 | 0.535 | 2.3 | 91.5 |
| sisVIVE - IPRS | 0.772 | 0.761 (0.140) | 0.058 | 0.944 | 0.1 | 99.4 |
| Two-sample strategy |  |  |  |  |  |  |
| True: ${\tilde{\boldsymbol{b}}}_{\boldsymbol{j}}\boldsymbol{=}\boldsymbol{\beta}_{\boldsymbol{j}}\boldsymbol{+}\boldsymbol{\theta}_{\boldsymbol{j}}$ |  |  |  |  |  |  |
| EPRS | 0.967 | 0.968 (0.080) | 0.031 | 1.371 | 0 | 100 |
| 2SLS | 0.969 | 0.970 (0.079) | 0.057 | 1.376 | 0 | 100 |
| Weighted Egger | 1.145 | 1.149 (0.104) | 0.057 | 1.830 | 0 | 100 |
| Weighted Median | 1.015 | 1.016 (0.116) | 0.066 | 1.492 | 0 | 100 |
| sisVIVE - SPRS | 0.547 | 0.550 (0.149) | 0.077 | 0.585 | 0.1 | 98.8 |
| sisVIVE - 2SLS | 0.823 | 0.823 (0.129) | 0.051 | 1.062 | 0 | 99.7 |
| Precise: ${\tilde{\boldsymbol{b}}}_{\boldsymbol{j}}\boldsymbol{\sim N(}\boldsymbol{\beta}_{\boldsymbol{j}}\boldsymbol{+}\boldsymbol{\theta}_{\boldsymbol{j}}\boldsymbol{,}\boldsymbol{0.01}^{\boldsymbol{2}}\boldsymbol{)}$ | |  |  |  |  |  |
| EPRS | 0.968 | 0.968 (0.081) | 0.031 | 1.371 | 0 | 100 |
| 2SLS | 0.960 | 0.960 (0.079) | 0.058 | 1.353 | 0 | 100 |
| Weighted Egger | 1.128 | 1.132 (0.101) | 0.059 | 1.784 | 0 | 100 |
| Weighted Median | 1.010 | 1.011 (0.117) | 0.066 | 1.480 | 0 | 100 |
| sisVIVE - SPRS | 0.534 | 0.529 (0.144) | 0.078 | 0.552 | 0.1 | 98.8 |
| sisVIVE - 2SLS | 0.817 | 0.814 (0.133) | 0.051 | 1.045 | 0 | 99.8 |
| Imprecise: ${\tilde{\boldsymbol{b}}}_{\boldsymbol{j}}\boldsymbol{\sim N(}\boldsymbol{\beta}_{\boldsymbol{j}}\boldsymbol{+}\boldsymbol{\theta}_{\boldsymbol{j}}\boldsymbol{,}\boldsymbol{0.05}^{\boldsymbol{2}}\boldsymbol{)}$ | |  |  |  |  |  |
| EPRS | 0.967 | 0.969 (0.087) | 0.035 | 1.374 | 0 | 100 |
| 2SLS | 0.777 | 0.773 (0.091) | 0.069 | 0.955 | 0 | 100 |
| Weighted Egger | 1.002 | 1.002 (0.114) | 0.064 | 1.459 | 0 | 100 |
| Weighted Median | 0.894 | 0.891 (0.139) | 0.078 | 1.210 | 0 | 100 |
| sisVIVE - SPRS | 0.097 | 0.112 (0.184) | 0.123 | 0.131 | 38.6 | 26.3 |
| sisVIVE - 2SLS | 0.302 | 0.352 (0.235) | 0.074 | 0.360 | 0.1 | 77.4 |

Table S10 Simulation results for multiple instruments (20 SNPs have positive direct and indirect pleiotropy with$\alpha_{s}\sim U\left( 0,0.2 \right), \theta_{s}\sim U(0, 0.4))$

| Methods | Median | Mean (SD) | Mean SE | MSE | Coverage % | Power % |
| --- | --- | --- | --- | --- | --- | --- |
| True value | **-0.2** |  |  |  |  |  |
| One-sample strategy |  |  |  |  |  |  |
| SPRS | 0.822 | 0.822 (0.061) | 0.034 | 1.049 | 0 | 100 |
| IPRS | 1.001 | 1.000 (0.056) | 0.045 | 1.444 | 0 | 100 |
| Weighted Egger | 1.093 | 1.097 (0.069) | 0.055 | 1.687 | 0 | 100 |
| Weighted Median | 1.009 | 1.007 (0.085) | 0.052 | 1.465 | 0 | 100 |
| sisVIVE - SPRS | 0.788 | 0.784 (0.094) | 0.069 | 0.976 | 0 | 99.9 |
| sisVIVE - IPRS | 0.931 | 0.933 (0.082) | 0.036 | 1.292 | 0 | 100 |
| Two-sample strategy |  |  |  |  |  |  |
| True: ${\tilde{\boldsymbol{b}}}_{\boldsymbol{j}}\boldsymbol{=}\boldsymbol{\beta}_{\boldsymbol{j}}\boldsymbol{+}\boldsymbol{\theta}_{\boldsymbol{j}}$ |  |  |  |  |  |  |
| EPRS | 1.018 | 1.018 (0.058) | 0.023 | 1.487 | 0 | 100 |
| 2SLS | 1.019 | 1.018 (0.057) | 0.045 | 1.488 | 0 | 100 |
| Weighted Egger | 1.130 | 1.132 (0.071) | 0.053 | 1.778 | 0 | 100 |
| Weighted Median | 1.016 | 1.019 (0.084) | 0.053 | 1.493 | 0 | 100 |
| sisVIVE - SPRS | 0.795 | 0.792 (0.114) | 0.062 | 0.997 | 0.1 | 99.9 |
| sisVIVE - 2SLS | 0.956 | 0.955 (0.094) | 0.039 | 1.342 | 0.1 | 100 |
| Precise: ${\tilde{\boldsymbol{b}}}_{\boldsymbol{j}}\boldsymbol{\sim N(}\boldsymbol{\beta}_{\boldsymbol{j}}\boldsymbol{+}\boldsymbol{\theta}_{\boldsymbol{j}}\boldsymbol{,}\boldsymbol{0.01}^{\boldsymbol{2}}\boldsymbol{)}$ | |  |  |  |  |  |
| EPRS | 1.018 | 1.018 (0.058) | 0.023 | 1.487 | 0 | 100 |
| 2SLS | 1.013 | 1.013 (0.057) | 0.046 | 1.474 | 0 | 100 |
| Weighted Egger | 1.122 | 1.123 (0.071) | 0.054 | 1.754 | 0 | 100 |
| Weighted Median | 1.013 | 1.015 (0.086) | 0.053 | 1.484 | 0 | 100 |
| sisVIVE - SPRS | 0.775 | 0.774 (0.113) | 0.062 | 0.961 | 0.1 | 99.9 |
| sisVIVE - 2SLS | 0.951 | 0.952 (0.096) | 0.039 | 1.335 | 0.1 | 100 |
| Imprecise: ${\tilde{\boldsymbol{b}}}_{\boldsymbol{j}}\boldsymbol{\sim N(}\boldsymbol{\beta}_{\boldsymbol{j}}\boldsymbol{+}\boldsymbol{\theta}_{\boldsymbol{j}}\boldsymbol{,}\boldsymbol{0.05}^{\boldsymbol{2}}\boldsymbol{)}$ | |  |  |  |  |  |
| EPRS | 1.019 | 1.018 (0.060) | 0.024 | 1.487 | 0 | 100 |
| 2SLS | 0.902 | 0.902 (0.064) | 0.058 | 1.218 | 0 | 100 |
| Weighted Egger | 1.060 | 1.061 (0.087) | 0.073 | 1.598 | 0 | 100 |
| Weighted Median | 0.945 | 0.944 (0.095) | 0.052 | 1.317 | 0 | 100 |
| sisVIVE - SPRS | 0.636 | 0.637 (0.153) | 0.074 | 0.707 | 0.6 | 98.1 |
| sisVIVE - 2SLS | 0.837 | 0.833 (0.104) | 0.044 | 1.079 | 0.1 | 99.9 |

Table S11 Simulation results for multiple instruments (30 SNPs have positive direct and indirect pleiotropy with$\alpha_{s}\sim U\left( 0,0.2 \right), \theta_{s}\sim U(0, 0.4))$

| Methods | Median | Mean (SD) | Mean SE | MSE | Coverage % | Power % |
| --- | --- | --- | --- | --- | --- | --- |
| True value | -0.2 |  |  |  |  |  |
| One-sample strategy |  |  |  |  |  |  |
| SPRS | 0.944 | 0.945 (0.052) | 0.027 | 1.313 | 0 | 100 |
| IPRS | 1.025 | 1.025 (0.048) | 0.041 | 1.502 | 0 | 100 |
| Weighted Egger | 1.080 | 1.080 (0.060) | 0.055 | 1.643 | 0 | 100 |
| Weighted Median | 1.017 | 1.014 (0.071) | 0.046 | 1.480 | 0 | 100 |
| sisVIVE - SPRS | 0.894 | 0.895 (0.078) | 0.053 | 1.206 | 0 | 100 |
| sisVIVE - IPRS | 0.988 | 0.987 (0.071) | 0.031 | 1.415 | 0 | 100 |
| Two-sample strategy |  |  |  |  |  |  |
| True: ${\tilde{\boldsymbol{b}}}_{\boldsymbol{j}}\boldsymbol{=}\boldsymbol{\beta}_{\boldsymbol{j}}\boldsymbol{+}\boldsymbol{\theta}_{\boldsymbol{j}}$ |  |  |  |  |  |  |
| EPRS | 1.038 | 1.037 (0.048) | 0.017 | 1.532 | 0 | 100 |
| 2SLS | 1.037 | 1.037 (0.048) | 0.041 | 1.533 | 0 | 100 |
| Weighted Egger | 1.112 | 1.111 (0.059) | 0.054 | 1.722 | 0 | 100 |
| Weighted Median | 1.025 | 1.025 (0.071) | 0.046 | 1.505 | 0 | 100 |
| sisVIVE - SPRS | 0.901 | 0.896 (0.089) | 0.053 | 1.210 | 0 | 100 |
| sisVIVE - 2SLS | 1.003 | 1.002 (0.072) | 0.036 | 1.450 | 0 | 100 |
| Precise: ${\tilde{\boldsymbol{b}}}_{\boldsymbol{j}}\boldsymbol{\sim N(}\boldsymbol{\beta}_{\boldsymbol{j}}\boldsymbol{+}\boldsymbol{\theta}_{\boldsymbol{j}}\boldsymbol{,}\boldsymbol{0.01}^{\boldsymbol{2}}\boldsymbol{)}$ | |  |  |  |  |  |
| EPRS | 1.036 | 1.037 (0.048) | 0.017 | 1.532 | 0 | 100 |
| 2SLS | 1.032 | 1.034 (0.048) | 0.042 | 1.524 | 0 | 100 |
| Weighted Egger | 1.104 | 1.105 (0.060) | 0.055 | 1.705 | 0 | 100 |
| Weighted Median | 1.020 | 1.023 (0.071) | 0.046 | 1.500 | 0 | 100 |
| sisVIVE - SPRS | 0.880 | 0.882 (0.093) | 0.053 | 1.179 | 0 | 100 |
| sisVIVE - 2SLS | 0.999 | 1.000 (0.075) | 0.036 | 1.447 | 0 | 100 |
| Imprecise: ${\tilde{\boldsymbol{b}}}_{\boldsymbol{j}}\boldsymbol{\sim N(}\boldsymbol{\beta}_{\boldsymbol{j}}\boldsymbol{+}\boldsymbol{\theta}_{\boldsymbol{j}}\boldsymbol{,}\boldsymbol{0.05}^{\boldsymbol{2}}\boldsymbol{)}$ | |  |  |  |  |  |
| EPRS | 1.036 | 1.036 (0.049) | 0.020 | 1.530 | 0 | 100 |
| 2SLS | 0.952 | 0.953 (0.057) | 0.052 | 1.333 | 0 | 100 |
| Weighted Egger | 1.062 | 1.061 (0.077) | 0.072 | 1.596 | 0 | 100 |
| Weighted Median | 0.962 | 0.962 (0.079) | 0.046 | 1.356 | 0 | 100 |
| sisVIVE - SPRS | 0.802 | 0.798 (0.122) | 0.058 | 1.012 | 0 | 99.9 |
| sisVIVE - 2SLS | 0.918 | 0.920 (0.081) | 0.039 | 1.262 | 0 | 100 |

Supplementary Section 2: Interpretation and Identification

S2.1 Interpretation of the ALICE causal model

Kang et al. [^1^](#_ENREF_1) assume that the ALICE model [^2^](#_ENREF_2) holds for the potential outcome $Y_{i}(d,\mathbf{z})$. The ALICE model comprises two components which can be written as follows:

$$E\left\{ Y_{i}\left( 0,\mathbf{0} \right)\left| \mathbf{Z}_{i}=\mathbf{z} \right. \right\}=\pi_{0}+\mathbf{z}^{'}\boldsymbol{\theta}\mathrm{and}Y_{i}\left( d,\mathbf{z} \right)-Y_{i}\left( 0,\mathbf{0} \right)=\mathbf{z}^{'}\boldsymbol{\alpha}+d\gamma_{X;i}, (S.1)$$

where the multiple instrumental variables $\mathbf{Z}_{i}$ are the SNPs, and $\mathbf{z}$ is one of the possible outcomes that this random vector can take. The exposure variable is $D_{i}$ which we take to be binary without loss of generality. The causal exposure effect for individual $i=1,\ldots,n$ is $\gamma_{X;i}$. The homogeneous-effects model is the special case of model $(S.1)$ with $\gamma_{X;i}=\gamma_{X}$.

Under the consistency assumption, the observed outcome is related to model (S.1) by $Y_{i}{=Y}_{i}(D_{i},\mathbf{Z}_{i})$. We can hence write

$$Y_{i}=Y_{i}\left( 0,0 \right)+\left\{ Y_{i}(D_{i},\mathbf{Z}_{i})-Y_{i}(0,\mathbf{0}) \right\}=\pi_{0}+\mathbf{Z}_{i}^{'}\left( \boldsymbol{\alpha}+\boldsymbol{\theta} \right)+D_{i}E\left( \beta_{i} \right)+D_{i}\left\{ \beta_{i}-E(\gamma_{X;i}) \right\}+\left\{ Y_{i}\left( 0,0 \right)-\pi_{0}-\mathbf{Z}_{i}^{'}\boldsymbol{\theta} \right\}\equiv\mathbf{Z}_{1i}^{'}\boldsymbol{\pi}+D_{i}E(\beta_{i})+D_{i}\left\{ \beta_{i}-E\left( \gamma_{X;i} \right) \right\}+\epsilon_{i}, (S.2)$$

where $\mathbf{Z}_{1i}=\left( 1,\mathbf{Z}_{i}^{'} \right)'$, $\boldsymbol{\pi}=\left( \pi_{0},\ldots,\pi_{J} \right)'$ and $\pi_{j}=\alpha_{j}+\theta_{j}$ for $j=1,\ldots,J$. In practice, sisVIVE is used with mean-centred outcomes and instruments so that $\pi_{0}=0$.

In the oracle 2SLS case, the identification of sisVIVE comes from the conditional moment restriction

$$E\left( \epsilon_{Yi}\left| \mathbf{Z}_{i} \right. \right)=0, (S.3)$$

which holds by construction for model $(S.2)$. This can be expanded under model $(S.2)$ as

$$E\left( \epsilon_{Yi}\left| \mathbf{Z}_{i} \right. \right)=E\left[ Y_{i}-\mathbf{Z}_{1i}^{'}\boldsymbol{\pi}-D_{i}E\left( \gamma_{X;i} \right)-D_{i}\left\{ \gamma_{X;i}-E\left( \gamma_{X;i} \right) \right\}\left| \mathbf{Z}_{i} \right. \right],$$

which reduces to

$$E\left( \epsilon_{Yi}\left| \mathbf{Z}_{i} \right. \right)=E\left( Y_{i}-\mathbf{Z}_{1i}^{'}\boldsymbol{\pi}-D_{i}\gamma_{X}\left| \mathbf{Z}_{i} \right. \right)\equiv E\left\{ r_{i}\left( \boldsymbol{\pi},\gamma_{X} \right)\left| \mathbf{Z}_{i} \right. \right\} (S.4)$$

under the homogeneous-effects assumption. If we want the heterogeneous-effects residual to have the same form as $r_{i}\left( \boldsymbol{\pi},\gamma_{X} \right)$ (where $\gamma_{X}=E\left( \gamma_{X;i} \right)$ is the average causal effect) then we need $E\left[ D_{i}\left\{ \gamma_{X;i}-E\left( \gamma_{X;i} \right) \right\}\left| \mathbf{Z}_{i} \right. \right]=0$ to hold.

The assumption given in Section 2.3 of Kang et al. [^1^](#_ENREF_1) is that there must be conditional independence between $\gamma_{X;i}-E\left( \gamma_{X;i} \right)$ and $D_{i}$ given $\mathbf{Z}_{i}$. This implies a zero covariance such that

$$E\left[ D_{i}\left\{ \gamma_{X;i}-E\left( \gamma_{X;i} \right) \right\}\left| \mathbf{Z}_{i} \right. \right]=E\left( D_{i}\left| \mathbf{Z}_{i} \right. \right)E\left\{ \gamma_{X;i}-E\left( \gamma_{X;i} \right)\left| \mathbf{Z}_{i} \right. \right\}.$$

However, this is generally non-zero unless $E\left\{ \gamma_{X;i}-E\left( \gamma_{X;i} \right)\left| \mathbf{Z}_{i} \right. \right\}=0$ because non-redundant IVs must satisfy $E\left( D_{i}\left| \mathbf{Z}_{i} \right. \right)\neq0$. Hence, we also require that $E\left( \gamma_{X;i}\left| \mathbf{Z}_{i} \right. \right)=E\left( \gamma_{X;i} \right)$, that is, the average treatment effect and IV must be uncorrelated. It follows that not only do we require a conditional correlation of zero (which they make clear), but also that $\gamma_{X;i}$ and $\mathbf{Z}_{i}$ are mean independent.

If this condition did not hold then it is possible that $E\left\{ \gamma_{X;i}-E\left( \gamma_{X;i} \right)\left| \mathbf{Z}_{i} \right. \right\}=\mathbf{Z}_{i}^{'}\boldsymbol{\eta}$, which would change the interpretation of $\mathbf{Z}_{i}^{'}\boldsymbol{\pi}$ in (S.4) to $\mathbf{Z}_{i}^{'}\boldsymbol{\pi}^{\mathrm{new}}$, where $\boldsymbol{\pi}^{\mathrm{new}}\boldsymbol{\equiv\pi}+\boldsymbol{\eta}$ and $\boldsymbol{\pi}^{\mathrm{new}}=\mathbf{0}$ does not correspond to the absence of pleiotropy.

Section S.2: Pleiotropy Scenario 3 where InSIDE fails but sisVIVE satisfies the Irrepresentable Condition of [^3^](#_ENREF_3).

From Corollary 1 of [^4^](#_ENREF_4): Lasso is consistent if $s<L-\left| {b_{1}}/{b_{2}} \right|g$ where $g=s$ if all total (direct plus indirect) pleiotropy effects are positive. Under indirect pleiotropy, $b_{1}=\beta+\theta$ for invalid SNPs and $b_{2}=\beta$ otherwise, so we need to choose $\theta<\left( L-2S \right)\beta/s$.

This leads the following simulation design:

- $Z_{1},\ldots,Z_{71}$ generated from trinomial distribution as before;
- $\beta_{j}=\beta=0.028$, j=1,…,71,
- $\alpha_{s}=\alpha=0.05$;
- For S=10, $\theta_{s}=\theta=0.1<\left( 71-2*10 \right)*\frac{0.028}{10}=0.1428$, s=1, …, 10
- For S=20, $\theta_{s}=\theta=0.02<\left( 71-2*20 \right)*\frac{0.028}{20}=0.0434$, s=1,…, 20
- For S=30, $\theta_{s}=\theta=0.005<\left( 71-2*30 \right)*\frac{0.028}{30}=0.0102$, s=1,…, 30
- $\alpha_{j}=0$ and $\theta_{j}=0$ if $j\neq s$;
- $X=\sum_{j}^{J} (\beta_{j}+\theta_{j})Z_{j}+U+\epsilon_{X};$
- $Y=\gamma X+\sum_{j}^{J} {(\alpha}_{j}+\theta_{j})Z_{j}+\gamma_{U}U+\epsilon_{Y};$
- $U, \epsilon_{X}$,$\epsilon_{Y}$ are independent and from $\sim N(0,1)$

Table S12 Monte Carlo estimates of FSO and FSI in simulated population

| No of invalid IVs | One sample  ${\hat{\boldsymbol{b}}}_{\boldsymbol{j}}\boldsymbol{=}{\hat{\boldsymbol{b}}}_{\boldsymbol{j}}$ | | Two sample  True: ${\tilde{\boldsymbol{b}}}_{\boldsymbol{j}}\boldsymbol{=}\boldsymbol{\beta}_{\boldsymbol{j}}$ | | Two sample  Precise:  ${\tilde{\boldsymbol{b}}}_{\boldsymbol{j}}\boldsymbol{\sim N(}\boldsymbol{\beta}_{\boldsymbol{j}}\boldsymbol{,}\boldsymbol{0.01}^{\boldsymbol{2}}\boldsymbol{)}$ | | Two sample  Imprecise:  ${\tilde{\boldsymbol{b}}}_{\boldsymbol{j}}\boldsymbol{\sim N(}\boldsymbol{\beta}_{\boldsymbol{j}}\boldsymbol{,}\boldsymbol{0.05}^{\boldsymbol{2}}\boldsymbol{)}$ | |
| --- | --- | --- | --- | --- | --- | --- | --- | --- |
|  | MFSI  (%) | MFSO  (%) | MFSI  (%) | MFSO  (%) | MFSI  (%) | MFSO  (%) | MFSI  (%) | MFSO  (%) |
| 10 | 20.8 | 37.9 | 63.9 | 21.2 | 54.3 | 23.8 | 5.2 | 16.0 |
| 20 | 48.8 | 21.3 | 75.3 | 13.8 | 68.6 | 14.6 | 30.5 | 13.0 |
| 30 | 67.4 | 17.1 | 82.5 | 13.9 | 76.9 | 15.2 | 45.9 | 13.4 |

Table S13 Simulation results for multiple instruments (10 SNPs have positive direct and indirect pleiotropy$)$ where InSIDE fails but sisVIVE satisfies the Irrepresentable Condition of [^3^](#_ENREF_3)

| Methods | Median | Mean (SD) | Mean SE | MSE | Coverage % | Power % |
| --- | --- | --- | --- | --- | --- | --- |
| True value | **-0.2** |  |  |  |  |  |
| One-sample strategy |  |  |  |  |  |  |
| SPRS | 0.316 | 0.317 (0.054) | 0.055 | 0.270 | 0 | 100.0 |
| IPRS | 0.659 | 0.661 (0.048) | 0.068 | 0.744 | 0 | 100.0 |
| Weighted Egger | 1.024 | 1.027 (0.077) | 0.086 | 1.511 | 0 | 100.0 |
| Weighted Median | 0.852 | 0.851 (0.069) | 0.088 | 1.110 | 0 | 100.0 |
| sisVIVE - SPRS | 0.283 | 0.273 (0.129) | 0.123 | 0.240 | 7.4 | 59.5 |
| sisVIVE - IPRS | 0.440 | 0.445 (0.158) | 0.091 | 0.442 | 0 | 93.9 |
| Two-sample strategy |  |  |  |  |  |  |
| True: ${\tilde{\boldsymbol{b}}}_{\boldsymbol{j}}\boldsymbol{=}\boldsymbol{\beta}_{\boldsymbol{j}}$ |  |  |  |  |  |  |
| EPRS | 0.719 | 0.719 (0.048) | 0.047 | 0.848 | 0 | 100.0 |
| 2SLS | 0.718 | 0.717 (0.045) | 0.072 | 0.843 | 0 | 100.0 |
| Weighted Egger | 1.299 | 1.298 (0.072) | 0.073 | 2.250 | 0 | 100.0 |
| Weighted Median | 0.922 | 0.923 (0.062) | 0.091 | 1.265 | 0 | 100.0 |
| sisVIVE - SPRS | 0.349 | 0.354 (0.098) | 0.070 | 0.316 | 0.5 | 96.2 |
| sisVIVE - 2SLS | 0.673 | 0.646 (0.118) | 0.072 | 0.730 | 0.1 | 98.6 |
| Precise: ${\tilde{\boldsymbol{b}}}_{\boldsymbol{j}}\boldsymbol{\sim N(}\boldsymbol{\beta}_{\boldsymbol{j}}\boldsymbol{,}\boldsymbol{0.01}^{\boldsymbol{2}}\boldsymbol{)}$ | |  |  |  |  |  |
| EPRS | 0.717 | 0.719 (0.050) | 0.048 | 0.848 | 0 | 100.0 |
| 2SLS | 0.692 | 0.695 (0.047) | 0.073 | 0.803 | 0 | 100.0 |
| Weighted Egger | 1.208 | 1.206 (0.079) | 0.079 | 1.984 | 0 | 100.0 |
| Weighted Median | 0.901 | 0.901 (0.066) | 0.091 | 1.216 | 0 | 100.0 |
| sisVIVE - SPRS | 0.328 | 0.325 (0.104) | 0.074 | 0.286 | 0.8 | 91.8 |
| sisVIVE - 2SLS | 0.630 | 0.601 (0.139) | 0.073 | 0.661 | 0.2 | 97.0 |
| Imprecise: ${\tilde{\boldsymbol{b}}}_{\boldsymbol{j}}\boldsymbol{\sim N(}\boldsymbol{\beta}_{\boldsymbol{j}}\boldsymbol{,}\boldsymbol{0.05}^{\boldsymbol{2}}\boldsymbol{)}$ | |  |  |  |  |  |
| EPRS | 0.723 | 0.726 (0.089) | 0.064 | 0.866 | 0 | 100.0 |
| 2SLS | 0.400 | 0.402 (0.056) | 0.069 | 0.365 | 0 | 100.0 |
| Weighted Egger | 0.590 | 0.590 (0.105) | 0.107 | 0.635 | 0 | 99.5 |
| Weighted Median | 0.478 | 0.444 (0.175) | 0.083 | 0.445 | 0.1 | 89.0 |
| sisVIVE - SPRS | -0.047 | -0.047 (0.112) | 0.103 | 0.035 | 64.6 | 7.9 |
| sisVIVE - 2SLS | 0.072 | 0.092 (0.106) | 0.056 | 0.097 | 0.7 | 33.6 |

Table S14 Simulation results for multiple instruments (20 SNPs have positive direct and indirect pleiotropy$)$ where InSIDE fails but sisVIVE satisfies the Irrepresentable Condition of [^3^](#_ENREF_3)

| Methods | Median | Mean (SD) | Mean SE | MSE | Coverage % | Power % |
| --- | --- | --- | --- | --- | --- | --- |
| True value | **-0.2** |  |  |  |  |  |
| One-sample strategy |  |  |  |  |  |  |
| SPRS | 0.427 | 0.431 (0.069) | 0.070 | 0.403 | 0 | 100.0 |
| IPRS | 0.529 | 0.532 (0.068) | 0.093 | 0.541 | 0 | 100.0 |
| Weighted Egger | 0.701 | 0.707 (0.170) | 0.182 | 0.851 | 0.3 | 97.2 |
| Weighted Median | 0.569 | 0.558 (0.152) | 0.121 | 0.597 | 0 | 98.1 |
| sisVIVE - SPRS | 0.319 | 0.311 (0.122) | 0.096 | 0.276 | 1.7 | 80.3 |
| sisVIVE - IPRS | 0.430 | 0.419 (0.127) | 0.085 | 0.400 | 0 | 96.5 |
| Two-sample strategy |  |  |  |  |  |  |
| True: ${\tilde{\boldsymbol{b}}}_{\boldsymbol{j}}\boldsymbol{=}\boldsymbol{\beta}_{\boldsymbol{j}}$ |  |  |  |  |  |  |
| EPRS | 0.624 | 0.628 (0.071) | 0.071 | 0.690 | 0 | 100.0 |
| 2SLS | 0.623 | 0.624 (0.068) | 0.111 | 0.683 | 0 | 100.0 |
| Weighted Egger | 3.284 | 3.291 (0.269) | 0.278 | 12.26 | 0 | 100.0 |
| Weighted Median | 0.873 | 0.873 (0.113) | 0.145 | 1.163 | 0 | 100.0 |
| sisVIVE - SPRS | 0.417 | 0.412 (0.105) | 0.080 | 0.386 | 0 | 97.5 |
| sisVIVE - 2SLS | 0.576 | 0.562 (0.119) | 0.101 | 0.594 | 0 | 99.3 |
| Precise: ${\tilde{\boldsymbol{b}}}_{\boldsymbol{j}}\boldsymbol{\sim N(}\boldsymbol{\beta}_{\boldsymbol{j}}\boldsymbol{,}\boldsymbol{0.01}^{\boldsymbol{2}}\boldsymbol{)}$ | |  |  |  |  |  |
| EPRS | 0.622 | 0.627 (0.079) | 0.074 | 0.690 | 0 | 100.0 |
| 2SLS | 0.573 | 0.578 (0.072) | 0.109 | 0.611 | 0 | 100.0 |
| Weighted Egger | 1.528 | 1.540 (0.229) | 0.272 | 3.080 | 0 | 100.0 |
| Weighted Median | 0.744 | 0.741 (0.133) | 0.141 | 0.904 | 0 | 99.9 |
| sisVIVE - SPRS | 0.386 | 0.374 (0.105) | 0.082 | 0.343 | 0.5 | 93.5 |
| sisVIVE - 2SLS | 0.519 | 0.501 (0.130) | 0.096 | 0.509 | 0.1 | 97.2 |
| Imprecise: ${\tilde{\boldsymbol{b}}}_{\boldsymbol{j}}\boldsymbol{\sim N(}\boldsymbol{\beta}_{\boldsymbol{j}}\boldsymbol{,}\boldsymbol{0.05}^{\boldsymbol{2}}\boldsymbol{)}$ | |  |  |  |  |  |
| EPRS | 0.640 | 0.645 (0.201) | 0.134 | 0.754 | 0.7 | 97.8 |
| 2SLS | 0.211 | 0.212 (0.066) | 0.074 | 0.174 | 0.1 | 84.1 |
| Weighted Egger | 0.209 | 0.204 (0.131) | 0.126 | 0.181 | 13.4 | 39.6 |
| Weighted Median | 0.162 | 0.167 (0.118) | 0.077 | 0.149 | 2.1 | 51.8 |
| sisVIVE - SPRS | 0.077 | 0.086 (0.143) | 0.099 | 0.102 | 28.6 | 25.6 |
| sisVIVE - 2SLS | 0.130 | 0.133 (0.101) | 0.054 | 0.121 | 1.0 | 60.9 |

Table S15 Simulation results for multiple instruments (30 SNPs have positive direct and indirect pleiotropy$)$ where InSIDE fails but sisVIVE satisfies the Irrepresentable Condition of [^3^](#_ENREF_3)

| Methods | Median | Mean (SD) | Mean SE | MSE | Coverage % | Power % |
| --- | --- | --- | --- | --- | --- | --- |
| True value | **-0.2** |  |  |  |  |  |
| One-sample strategy |  |  |  |  |  |  |
| SPRS | 0.586 | 0.591 (0.083) | 0.083 | 0.633 | 0 | 100.0 |
| IPRS | 0.527 | 0.530 (0.077) | 0.103 | 0.539 | 0 | 100.0 |
| Weighted Egger | 0.467 | 0.470 (0.192) | 0.199 | 0.486 | 7.6 | 65.4 |
| Weighted Median | 0.511 | 0.505 (0.154) | 0.128 | 0.521 | 0 | 94.7 |
| sisVIVE - SPRS | 0.474 | 0.471 (0.134) | 0.099 | 0.468 | 0 | 95.8 |
| sisVIVE - IPRS | 0.483 | 0.475 (0.115) | 0.093 | 0.469 | 0 | 99.0 |
| Two-sample strategy |  |  |  |  |  |  |
| True: ${\tilde{\boldsymbol{b}}}_{\boldsymbol{j}}\boldsymbol{=}\boldsymbol{\beta}_{\boldsymbol{j}}$ |  |  |  |  |  |  |
| EPRS | 0.656 | 0.660 (0.085) | 0.085 | 0.757 | 0 | 100.0 |
| 2SLS | 0.654 | 0.655 (0.080) | 0.129 | 0.738 | 0 | 100.0 |
| Weighted Egger | 10.75 | 10.78 (1.015) | 1.035 | 121.6 | 0 | 100.0 |
| Weighted Median | 0.752 | 0.752 (0.133) | 0.167 | 0.925 | 0 | 99.9 |
| sisVIVE - SPRS | 0.576 | 0.578 (0.115) | 0.093 | 0.621 | 0 | 99.9 |
| sisVIVE - 2SLS | 0.636 | 0.632 (0.116) | 0.116 | 0.705 | 0 | 99.9 |
| Precise: ${\tilde{\boldsymbol{b}}}_{\boldsymbol{j}}\boldsymbol{\sim N(}\boldsymbol{\beta}_{\boldsymbol{j}}\boldsymbol{,}\boldsymbol{0.01}^{\boldsymbol{2}}\boldsymbol{)}$ | |  |  |  |  |  |
| EPRS | 0.652 | 0.660 (0.097) | 0.090 | 0.749 | 0 | 100.0 |
| 2SLS | 0.585 | 0.592 (0.085) | 0.124 | 0.634 | 0 | 100.0 |
| Weighted Egger | 0.625 | 0.635 (0.379) | 0.392 | 0.841 | 44.9 | 36.9 |
| Weighted Median | 0.612 | 0.608 (0.162) | 0.157 | 0.679 | 0.2 | 96.5 |
| sisVIVE - SPRS | 0.551 | 0.542 (0.122) | 0.094 | 0.566 | 0.1 | 99.2 |
| sisVIVE - 2SLS | 0.565 | 0.564 (0.124) | 0.109 | 0.599 | 0.1 | 99.2 |
| Imprecise: ${\tilde{\boldsymbol{b}}}_{\boldsymbol{j}}\boldsymbol{\sim N(}\boldsymbol{\beta}_{\boldsymbol{j}}\boldsymbol{,}\boldsymbol{0.05}^{\boldsymbol{2}}\boldsymbol{)}$ | |  |  |  |  |  |
| EPRS | 0.667 | 0.699 (0.402) | 0.248 | 0.969 | 3.3 | 92.0 |
| 2SLS | 0.178 | 0.179 (0.070) | 0.076 | 0.149 | 0.2 | 67.3 |
| Weighted Egger | 0.145 | 0.149 (0.133) | 0.129 | 0.140 | 23.1 | 23.2 |
| Weighted Median | 0.154 | 0.157 (0.110) | 0.079 | 0.139 | 2.7 | 51.0 |
| sisVIVE - SPRS | 0.227 | 0.233 (0.160) | 0.105 | 0.213 | 11.1 | 56.0 |
| sisVIVE - 2SLS | 0.164 | 0.159 (0.100) | 0.056 | 0.139 | 0.8 | 70.3 |

S2.3 Identification with indirect pleiotropy and failure of InSIDE

Consider oracle 2SLS fitting of model (6) from our paper, that is, the identities of the valid-IV and invalid-IV SNPs are known. We then proceed by contradiction: suppose that Theorem 1 is satisfied and $\gamma_{X}$ is identified. Ignoring the constant term, model (S.1) directly implies that

$$E\left( Y | \mathbf{Z} \right)=\mathbf{Z}^{'}\boldsymbol{\pi}+E\left( D | \mathbf{Z} \right)\gamma_{X}. (S.5)$$

The identification of $\gamma_{X}$ comes through the additional assumption that

$$E\left( D | \mathbf{Z} \right)=b_{0}+\sum_{j=1}^{J} b_{j}Z_{j}, (S.6)$$

where our simulation design coupled with orthogonal SNPs give us that $b_{j}=\beta_{j}+\theta_{j}$ if $Z_{j}$ is not valid, and $b_{j}=\beta_{j}$ if it is. (We can interpret $\beta_{j}$ as the direct/causal effect of $Z_{j}$ on the exposure, and $\theta_{j}$ as its indirect effect via the unobserved confounding variables.)

Combining (S.5) and (S.6) gives

$$E\left( Y | \mathbf{Z} \right)=b_{0}+\sum_{j=1}^{J} \left\{ \alpha_{j}+\theta_{j}+(\beta_{j}+\theta_{j})\gamma_{X} \right\}Z_{j}\equiv b_{0}+\sum_{j=1}^{J} a_{j}Z_{j}, (S.7)$$

so the data fix $\hat{b}_{j}=\beta_{j}+\theta_{j}$ and

$$\hat{a}_{j}=\alpha_{j}+\theta_{j}+(\beta_{j}+\theta_{j})\gamma_{X}.$$

Because $\theta_{j}=\hat{b}_{j}-\beta_{j}$, the right-hand side above can be rewritten as

$$\alpha_{j}+\theta_{j}\left( 1+\gamma_{X} \right)+\beta_{j}\gamma_{X}=\alpha_{j}+\hat{b}_{j}\left( 1+\gamma_{X} \right)-\beta_{j},$$

which still equals $\hat{a}_{j}$. Both $\hat{b}_{j}$ and $\gamma_{X}$ are fixed, but any values $\alpha_{j}\neq\alpha_{j}^{*}$ and $\beta_{j}^{*}\neq\beta_{j}$ satisfying $\alpha_{j}^{*}-\beta_{j}^{*}=\alpha_{j}-\beta_{j}$ still satisfy the data constraints.

Thus, with indirect pleiotropy, $\pi_{j}$ is not identified (among the invalid SNPs) because one can always find $\pi_{j}^{*}=\alpha_{j}^{*}+\theta_{j}\neq\pi_{j}$ subject to the above. This contradicts our initial assertion because, as Kang et al. [^1^](#_ENREF_1) pointed out (on page 136),

“Theorem 1 is a statement about uniqueness of solutions for the parameters [$\boldsymbol{\pi}$], and [$\gamma_{X}$] in Equation (7) … In the proof of Theorem 1, we show that … the parameter [$\gamma_{X}]$ is a unique solution to (7) **if and only if** the parameter [$\boldsymbol{\pi}$] is a unique solution to (7)”

where their equation (7) is equation (6) in our paper. In other words, if the solution to (S.5) is not unique then a unique solution cannot exist to the *consistency criterion* in Theorem 1.

Conversely, if there were no indirect pleiotropy in the simulation model, $\hat{a}_{j}=\alpha_{j}+\beta_{j}\gamma_{X}$ and $\hat{b}_{j}=\beta_{j}$ would uniquely identify $\gamma_{j}$ and hence $\pi_{j}=\alpha_{j}$. Similarly, if the simulation model held and $\beta_{j}$ were known instead of$b_{j}=\beta_{j}+\theta_{j}$, then unique $\alpha_{j}$ and $\theta_{j}$ could be found and the consistency criterion would hold. This fits with our findings (not presented) in the simulation study in which knowledge of the direct effect of SNP on exposure led to greatly improved sisVIVE performance.

References for supplementary

**1.** Kang H, Zhang A, Cai TT, Small DS. Instrumental Variables Estimation With Some Invalid Instruments and its Application to Mendelian Randomization. *JASA.* 2016;111(513):132-144.

**2.** Holland PW. Causal Inference, Path Analysis, and Recursive Structural Equations Models. *Sociological Methodology.* 1988;18(449-484).

**3.** Zou H. The adaptive lasso and its oracle properties. *JASA.* Dec 2006;101(476):1418-1429.

**4.** Windmeijer F, Farbmacher H, Davies N, Smith GD. On the use of the Lasso for instrumental variables estimation with some invalid instruments. 2017; available at: <http://www.efm.bris.ac.uk/economics/working_papers/pdffiles/dp16674.pdf>.
